# Exploring and Validating the Molecular Mechanisms Linking Fatty Acid Metabolism and Sarcopenia

**DOI:** 10.1049/syb2.70052

**Published:** 2025-12-29

**Authors:** Ruopeng Yang, Shan Gu, Yang Li, Ping Xia

**Affiliations:** ^1^ Hubei University of Chinese Medicine Wuhan China; ^2^ Department of Orthopaedics Traditional Chinese and Western Medicine Hospital Hubei University of Chinese Medicine Wuhan China; ^3^ Department of Orthopaedics Wuhan No.1 Hospital Wuhan China; ^4^ Department of Anesthesiology, Hubei Provincial Hospital of Traditional Chinese Medicine, Hubei Key Laboratory of Theory and Application Research of Liver and Kidney in Traditional Chinese Medicine (Hubei Province Hospital of Traditional Chinese Medicine) Affiliated Hospital of Hubei University of Chinese Medicine Wuhan China; ^5^ Department of Orthopedic Surgery, Hubei Provincial Hospital of Traditional Chinese Medicine, Hubei Key Laboratory of Theory and Application Research of Liver and Kidney in Traditional Chinese Medicine (Hubei Province Hospital of Traditional Chinese Medicine), Hubei Shizhen Laboratory Affiliated Hospital of Hubei University of Chinese Medicine Wuhan China; ^6^ Department of Orthopaedics Wuhan No.4 Hospital Wuhan China

**Keywords:** bioinformatics, fatty acid metabolism, muscle

## Abstract

Sarcopenia is an ageing‐related disease characterised primarily by skeletal muscle functional decline. Despite of fatty acid metabolism (FAM) affecting oxidative stress within muscle tissue, the key roles of critical genes linking FAM and sarcopenia are unclear. The GSE8479, GSE1428, and GSE136344 datasets were downloaded and intersected for identifying FAM‐related differentially expressed genes (FAMRDEGs) screened by enrichment analysis, LASSO regression, and Support Vector Machine (SVM) analyses. Cytoscape software was used for visualising mRNA‐transcription factor (TF) and mRNA‐miRNA networks. In addition, ROC curves of key genes were plotted to evaluate their diagnostic significance. A Fatty Acid Metabolism Score (FAM‐Score) was conducted and immune cell infiltration analysis was conducted. The qPCR assay was performed to analyse the levels of screened critical genes. A total of 109 FAMRDEGs were obtained, and the LASSO regression and SVM models screened 14 of these genes. The network included 7 key genes with 54 miRNAs and 9 hub genes with 102 TFs. There were 6 types of immune cell infiltration showing statistical significance. The *FABP3* (*P* < 0.001), *PECR* (*P* < 0.01), and *OPN3* (*P* < 0.001) mRNA expression markedly increased in sarcopenia versus control groups. In contrast, sarcopenia group showed remarkably reduced *PCTP* (*P* < 0.001), *SREBF2* (*P* < 0.001), and *PPARGC1A* (*P* < 0.05) levels. This study provides reference indicators for FAM‐associated auxiliary biomarkers of sarcopenia and preliminarily establishes effective machine learning models for further mechanistic exploration.

## Graphical abstract

This study provides reference for fatty acid metabolism related potential candidate genes for sarcopenia and initially creates an effective machine learning models for further investigating the direction of the mechanism level.

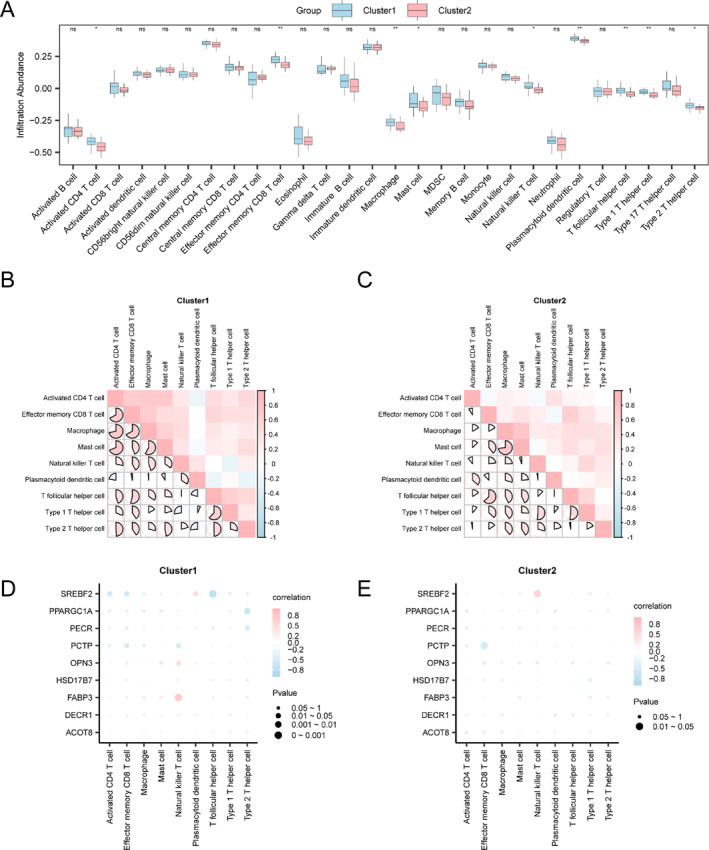

## 1 Introduction

Sarcopenia is a progressive disorder characterised primarily by skeletal muscle functional decline, accompanied by accelerated muscle mass loss and dysfunction, which in turn elevates the risk of falls, fractures, and ultimately mortality [1, 2, 3]. Furthermore, the incidence of cardiovascular events is increased by about 33% compared with healthy individuals [4, 5]. However, treatment options for sarcopenia remain limited. Although resistance training has been demonstrated to be an effective intervention, its clinical applicability is restricted, particularly among elderly patients. Consequently, current treatment strategies cannot adequately reduce sarcopenia risk, underscoring the urgent need for novel therapeutic approaches.

Fatty acid metabolism (FAM) is a key process in energy supply, cell signalling, and immune regulation. Recent studies have revealed its central role in immunometabolic homoeostasis, metabolic engineering, and multiple diseases [6]. Dysregulated FAM participates in diverse pathogenic processes through mechanisms such as inflammatory factor secretion, increased oxidative stress, and disrupted energy metabolism. Prior evidence suggests that excessive fatty acid synthesis exacerbates inflammation in autoimmune diseases, whereas impaired fatty acid oxidation (FAO) induces energetic crises in renal disorders [7, 8]. In sarcopenia, FAM dysregulation manifests as reduced FAO efficiency, branched‐chain amino acid (BCAA) accumulation, and mitochondrial dysfunction, collectively triggering skeletal muscle energy deficiency and atrophy. Infiltration of free fatty acids (FFAs) during chronic inflammation further aggravates oxidative stress within muscle tissue [9]. Nonetheless, the molecular network and metabolic interactions of FAM in sarcopenia still lack systematic investigation, which demands further elucidation through integrated multi‐omics strategies. We identified key genes by integrating GEO datasets to conduct differential analysis, Gene Ontology (GO) and Kyoto Encyclopaedia of Genes and Genomes (KEGG) mapping, and machine learning model construction, followed by reliability verification using multiple algorithms to determine effects of FAM‐related genes (FAMRGs) on sarcopenia. The identification of pivotal genes and regulatory pathways of FAM provides a foundation for understanding the molecular mechanisms underlying sarcopenia.

## 2 Materials and Methods

### 2.1 Data Collection

Sarcopenia related transcriptome chip datasets were screened from the GEO database [10] according to the following criteria: (1) the study species was limited to *Homo sapiens*; (2) the tissue source was the lateral thigh muscles; (3) the dataset contained both sarcopenia and healthy control groups; (4) the data type was mRNA expression profile chip data; (5) adequate sample size, complete grouping information, and traceable chip platform annotations. These criteria were applied to ensure homogeneity and comparability of data sources, reduce heterogeneity, and enhance the reliability of subsequent differential analysis and model construction.

In accordance with the specified inclusion criteria, three datasets, namely GSE8479 [11], GSE1428 [12], and GSE136344 [13], were ultimately obtained based on GEO database (https://www.ncbi.nlm.nih.gov/geo/). All three datasets were acquired from human lateral thigh muscles, included both sarcopenia and control samples, and were sequenced using platforms (GPL2700, GPL96, and GPL5175, respectively) with good compatibility and comparability. These datasets have been repeatedly applied in prior studies of muscle ageing and metabolism and demonstrate stable data quality, low background noise, and high representativeness, thereby supporting robust cross‐cohort integration. Detailed information is provided in Table 1.

**TABLE 1:** Overview of GEO microarray data.

	GSE8479	GSE1428	GSE136344
Platform	GPL2700	GPL96	GPL5175
Species	*Homo sapiens*	*Homo sapiens*	*Homo sapiens*
Tissue	Lateral thigh muscle tissue	Lateral thigh muscle tissue	Lateral thigh muscle tissue
Sarcopenia samples	25	12	12
Control samples	26	10	11
Reference	PMID:17520024	PMID:15687482	PMID:33921590

Abbreviation: GEO, Gene Expression Omnibus.

Probe annotation information for these platforms (GPL2700, GPL96, GPL5175) was obtained from the official GEO annotation files and standardised based on Gene Symbols corresponding to each probe. To reduce platform‐related bias, the average expression value of many probes linked with a single gene was used as the final expression level. All chip data were subjected to background‐correction and normalisation with the RMA (Robust Multi‐array Average) function of R package limma [14] (version 3.58.1). For reducing technical bias, principal component analysis (PCA) [15] was used to detect and remove abnormal samples, followed by ComBat batch effect correction using the R package sva [16] to ensure cross‐platform comparability. After quality control, a pooled dataset of 37 sarcopenia samples and 36 control samples was obtained for further analysis.

FAMRGs were acquired based on GeneCards database [17] (https://www.genecards.org/). FAMRGs represent integrative human gene data within the GeneCards database. A total of 342 FAMRGs were identified through database search using the keyword ‘fatty acid metabolism’ upon criteria of Relevance Score > 2 and protein‐coding classification. In addition, ‘Fatty Acid Metabolism’ was the keyword in PubMed (https://pubmed.ncbi.nlm.nih.gov/) for retrieving FAMRGs reported in the published literature [18], yielding 49 genes. After merging and deduplication, 367 FAMRGs were retained (see Table S1 for details).

### 2.2 Sarcopenia‐Related Fatty Acid Metabolism‐Related Differentially Expressed Genes (FAMRDEGs)

We categorised cohorts in pooled GEO datasets in the sarcopenia group or the control group based on sample annotations. By employing R package limma (version 3.58.1), differentially expressed genes (DEGs) were identified upon adjusted *p* value < 0.05 (Benjamini–Hochberg (BH) method), considering genes with |logFC| > 0. DEG outcomes were visualised using R package ggplot2 (version 3.4.4). Sarcopenia‐related FAMRDEGs were obtained by intersecting DEGs from the pooled datasets with FAMRGs, resulting in a Venn diagram to define FAMRDEGs. The top 20 FAMRDEGs were presented in the heatmap with R package pheatmap (version 1.0.12), selected based on ascending *p* values and descending |logFC| after BH correction to emphasise significantly altered expression patterns.

### 2.3 Functional Enrichment Analyses

FAMRDEGs underwent GO as well as KEGG enrichment for exploring the functional implications. GO annotation [19] covers biological process (BP), cellular component (CC), and molecular function (MF) categories, whereas KEGG [20] provides a repository of biological pathways and associated databases. These analyses were conducted using the clusterProfiler R package (version 4.10.0) [21], with terms considered significantly enriched at a threshold of *P* < 0.05 and false discovery rate (FDR) *q*‐value < 0.05, following BH adjustment for multiple testing.

### 2.4 Gene Set Enrichment Analysis (GSEA)

We implemented GSEA [22] to detect significantly associated predefined gene sets in the sarcopenia phenotype. By adopting R package clusterProfiler (version 4.10.0), each gene was ranked by the logFC value using the C2 curated gene sets from MSigDB (version 2023.2.Hs). Significant sets were determined through adjusted *p* value and FDR thresholds of < 0.05, with gene set sizes filtered between 10 and 500.

### 2.5 Sarcopenia Diagnostic Model Establishment

We constructed the sarcopenia diagnostic model using logistic regression on FAMRDEGs identified from the integrated dataset. First, 109 FAMRDEGs were assessed through univariate logistic regression (*P* < 0.05) to identify genes significantly associated with sarcopenia. After obtaining 103 candidate genes, these were subjected to LASSO regression and SVM‐based feature selection. R package glmnet [23] was utilised for LASSO regression using family = ‘binomial’, seed = 500, and 10‐fold cross‐validation for identifying the best penalty coefficient *λ* while reducing overfitting. Meanwhile, a SVM model was constructed using the e1071 package with a radial basis function kernel, and the optimal gene set was selected by comparing classification accuracy and error rates across different feature subsets. The intersecting genes identified by both LASSO and SVM were designated as key FAMRDEGs and used to construct a multifactor logistic regression model for diagnostic evaluation. In this way, the analytical relationship among logistic regression, LASSO, and SVM was clarified, and the modelling process was made more transparent and reproducible.

The results of the LASSO regression were presented in a diagnostic model plot and a variable coefficient trajectory plot. Using the regression coefficients from the model, we then calculated a LASSO Risk Score (RiskScore) for each sample with the following formula:

$$\text{RiskScore}=\sum_{i}\text{Coefficient}\;\left({\text{gene}}_{i}\right)\times \text{mRNA}\;\text{Expression}\;\left({\text{gene}}_{i}\right).$$

Subsequently, we constructed the SVM [24] algorithm through incorporating FAMRDEGs from the logistic regression model, so as to identify the feature subset that yielded the most accurate prediction whereas smallest error rate. For enhancing feature selection robustness, the overlapping genes that were consistently selected by both algorithms were retained for subsequent analysis.

### 2.6 Verification of the Sarcopenia Diagnostic Model

The rms package facilitated the development of a nomogram for the chosen FAMRDEGs. This nomogram uses aligned axes within a two‐dimensional coordinate system to depict the relationships among variables. Within the multifactor regression framework, each variable was allocated a specific scale to measure its contribution to risk, with the cumulative score serving to estimate the likelihood of the outcome event occurring.

A calibration curve, a method particularly suitable for logistic regression‐based models, was used for assessing the agreement of model‐predicted versus actual probabilities. Furthermore, the variables remained in multifactor regression model were key genes. Subsequently, the model's clinical applicability was evaluated by decision curve analysis (DCA), with R package ggDCA utilised for generating corresponding plots for assessing diagnostic benefit.

Besides, we utilised R package pROC (version 1.18.5) for constructing ROC curves, with Area Under the Curve (AUC) being determined. When the AUC fell between 0.5 and 1, the logistic regression model was considered to have acceptable diagnostic performance. A value approaching 1 indicated stronger discriminatory ability.

### 2.7 Protein–Protein Interaction (PPI) Network Establishment

PPI networks reflect the protein‐protein biological associations. Known and predicted interactions were queried in STRING database [25, 26]. During PPI network establishment, the species was limited to *Homo sapiens*, meanwhile, the confidence score ≥ 0.150 for key gene pairs was applied. Cytoscape was then used for visualisation. We used the GeneMANIA platform [27] to predict genes with similar functions and construct the final interaction network.

### 2.8 Regulatory Network Establishment

To investigate the relationships between significant genes and their associated miRNAs, we searched TarBase database [28] (http://www.microrna.gr/tarbase) to identify relevant miRNAs. Subsequently, Cytoscape was employed for visualising the mRNA‐miRNA network [29].

Transcription factors (TFs) mediate post‐transcriptional regulation of gene levels through interacting with key genes. In this study, TFs were acquired based on ChIPBase database [30] (http://rna.sysu.edu.cn/chipbase/), whereas the impacts on the key gene regulatory functions were analysed. Additionally, mRNA‐TF network visualisation was completed with Cytoscape.

### 2.9 Differential Expression, Correlation, and ROC Curve Analyses

To evaluate key DEGs in sarcopenia versus control groups, we created comparative plots according to each identified key gene level. Their diagnostic performance was subsequently assessed through ROC curve analysis with pROC package, and AUC values were determined to quantify their predictive power. AUC values of 0.5–0.7, 0.7–0.9, and > 0.9 suggest low, moderate, and high accuracy separately. Furthermore, the pooled GEO datasets were subjected to Spearman correlation analysis to assess correlations among key genes. The resulting correlation matrix was displayed by the heatmap with the pheatmap package (version 1.0.12). Specifically, |*R*‐values| < 0.3, 0.3–0.5, 0.5–0.8, and > 0.8 denote weak/no, weak, medium, and strong correlations, respectively.

### 2.10 Establishment of High‐Low Fatty Acid Metabolism Rating Groups

Single‐sample gene set enrichment analysis (ssGSEA) [31] was carried out for quantifying FAM‐related gene expression at the individual sample level. The FAM‐Scores of all samples were determined using R package GSVA, based on the pooled dataset‐derived key gene expression matrix. Group comparisons were visualised with ggplot2 in R, whereas ROC curves for the pooled datasets were plotted with pROC, meanwhile, AUC values were determined for evaluating FAM‐Score's diagnostic significance.

The median FAM‐Score was utilised to classify sarcopenia patients in HighScore or LowScore group, and KEGs expression was visualised by generating a comparative plot. Additionally, by employing pROC (version 1.18.5), ROC curves of FAM‐Score were obtained, with AUCs of key genes in sarcopenia samples being calculated. AUCs were rated as 0.5–0.7, 0.7–0.9, and > 0.9, representing low, moderate, as well as high diagnostic performance separately.

### 2.11 Immune Infiltration Analysis (ssGSEA)

ssGSEA was conducted quantifying various immune cell infiltration abundances, like Gamma‐delta T cells, activated CD8+ T cells, natural killer cells, activated dendritic cells, and regulatory T cells. This analysis generated an immune infiltration matrix across pooled datasets to derive enrichment scores for each immune cell subset in every sample. Using ggplot2 package (version 3.4.4), we visualised the differential infiltration patterns between the two disease groups, identifying several significantly altered immune cell types. Furthermore, Spearman correlation analysis was completed for evaluating interactions among infiltrating immune cells and key gene‐immune subset associations. The pheatmap package was utilised to visualise intercellular associations, whereas the ggplot2 package was adopted for generating bubble plots to depict gene‐immune cell relationships.

### 2.12 Construction of Sarcopenia Subtypes

To identify distinct disease subtypes based on key gene expression, we performed consensus clustering with R package ConsensusClusterPlus (version 1.72.0) [32, 33]. The resampling‐based method assesses the stability of cluster assignments. The cluster number was 2–9, distance metric was ‘euclidean’, and the clustering algorithm used was ‘pam’. In addition, 80% of all samples were resampled 50 times. The key DEGs across sarcopenia subtypes was visualised in a heatmap and subsequently validated through group comparison plots.

### 2.13 Primary Cell Culture

C2C12 mouse myoblasts were resuscitated in DMEM that contained 10% foetal bovine serum before culture within complete medium at 37°C, 5% CO_2_. After reaching about 90% confluence, the cells were added into differentiation medium that contained 2% horse serum for 48 h. Those cultured C2C12 cells were then divided into sarcopenia and control groups. The control group continued standard culture, whereas the sarcopenia group was treated with 40 g/L D‐galactose (D‐gal) to induce cell senescence [34].

### 2.14 Quantitative Real‐Time Polymerase Chain Reaction (qPCR)

Through utilising TRIzol reagent (TSINGKE, TSP401), total RNA was isolated in C2C12 mouse myoblasts following standard protocols, and later prepared into cDNA with SynScript III RT SuperMix for qPCR (TSINGKE, TSK314M) via reverse‐transcription. Quantitative detection of *PCTP*, *FABP3*, *PECR*, *SREBF2*, *PPARGC1A*, *ACOT8*, *DECR1*, *OPN3*, and *HSD17B7* was conducted using ArtiCanATM SYBR qPCR Mix (TSINGKE, TSE501) on a ViiA 7 qPCR detection system (ABI). PCR thermocycling conditions consisted of 1 min under 95°C; 10 s under 95°C and 20 s under 60°C for fluorescence collection over 40 cycles. mRNA expression was quantified by 2^–ΔΔCT^ method and GAPDH was the control. Each sample was analysed in triplicate. Primer sequences are provided in Table 2.

**TABLE 2:** Primer sequence.

Name	Sequence (5′‐3′)	Size
Mus GAPDH	TGTTTCCTCGTCCCGTAGA	116bp
	GATGGCAACAATCTCCACTTTG	
Mus PCTP	ATGGTGGCATACTGGGAAGT	171bp
	TTTCACTCGGATGACCCCAG	
Mus FABP3	TAGTGGACAGCAAGAATTTTGA	267bp
	CGTTCCACTTCTGCACATGGAT	
Mus PECR	GCGGCAAGAGAAGGTGTTTA	171bp
	TGGGATACTGTCAAAGGCCA	
Mus SREBF2	CTCCTCCTGTGGCTGGTAAA	162bp
	AGCTGCGAAATCACCTTTGG	
Mus PPARGC1A	GATGTGTCGCCTTCTTGCTC	230bp
	CGGTGTCTGTAGTGGCTTGA	
Mus ACOT8	GAAGTATCGAGTGGGGCTGAA	237bp
	TTATACTTGGACTGGTGGGGC	
Mus DECR1	CAGTCTAAATTCTTCCAGCCCG	159bp
	ATTTCTGCTGGCTATCACACAC	
Mus OPN3	GTTTCCATTACCACCCTCACTG	184bp
	AGCCCAGTCCATGTATGTCTAG	
Mus HSD17B7	GCAGAGGAAGTCAAGCAAAAGT	161bp
	TTCTGGGTCAAAATTCCTTCCG	

### 2.15 Statistical Analysis

Data were processed and analysed with R software (version 4.2.2). Without specific indications, normally‐distributed continuous data were subjected to independent Student's t‐test or Kruskal–Wallis test for assessments involving two or multiple groups. Conversely, non‐normally‐distributed data were examined through Mann–Whitney *U* test or the Wilcoxon rank sum test. Spearman correlation coefficients were employed for determining relationships among the variables. Experiment results were represented by mean ± standard deviation (SD) and analysed with GraphPad Prism 8.0 (GraphPad Software, USA). To ascertain between‐group statistical significance, we additionally conducted a two‐tailed *t*‐test, and *p* < 0.05 suggested statistical significance.

## 3 Results

### 3.1 Technical Flowchart

**FIGURE 1:**
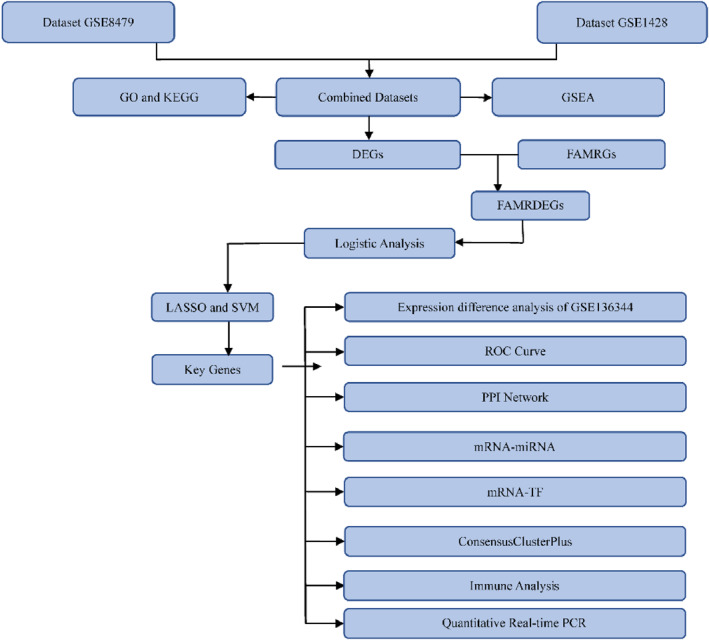
Technical flowchart for the analysis of FAMRDEGs. DEGs, Differentially Expressed Genes; FAMRDEGs, Fatty Acid Metabolism‐Related Differentially Expressed Genes; FAMRGs, Fatty Acid Metabolism‐Related Genes; GO, Gene Ontology; GSEA, Gene Set Enrichment Analysis; KEGG, Kyoto Encyclopaedia of Genes and Genomes; LASSO, Least Absolute Shrinkage and Selection Operator; PPI, Protein–Protein Interaction; ROC, Receiver Operating Characteristic; SVM, Support Vector Machine; TF, Transcription Factor.

To enhance the clarity of the research methodology, we have drawn a technical flowchart in Figure 1. This investigation amalgamated the GSE8479, GSE1428, and GSE136344 datasets and employed the sva package to eliminate batch effects. Following this, we utilised the limma package to perform differential expression analysis, leading to the identification of FAMRDEGs. To further investigate the biological roles of these genes, we executed Gene Ontology (GO) and Kyoto Encyclopaedia of Genes and Genomes (KEGG) enrichment analyses, along with constructing a Protein‐Protein Interaction (PPI) network through the STRING database. Moreover, Gene Set Enrichment Analysis (GSEA) unveiled significant signalling pathways, whereas Receiver Operating Characteristic (ROC) curve analysis was conducted to evaluate the diagnostic potential of the identified genes. mRNA‐miRNA and mRNA‐TF networks were constructed to screen associated miRNAs and TFs. The ConsensusClusterPlus was used to construct sarcopenia subtype and the ssGSEA algorithm was implemented to assess immune cell infiltration. Ultimately, we performed qPCR assay to identify the expression levels of 9 key genes. This technical flowchart supports the systematic of our study, thereby offering a solid foundation for the identification of prospective biomarkers and mechanisms associated with sarcopenia.

### 3.2 Data Collection and Normalisation

**FIGURE 2:**
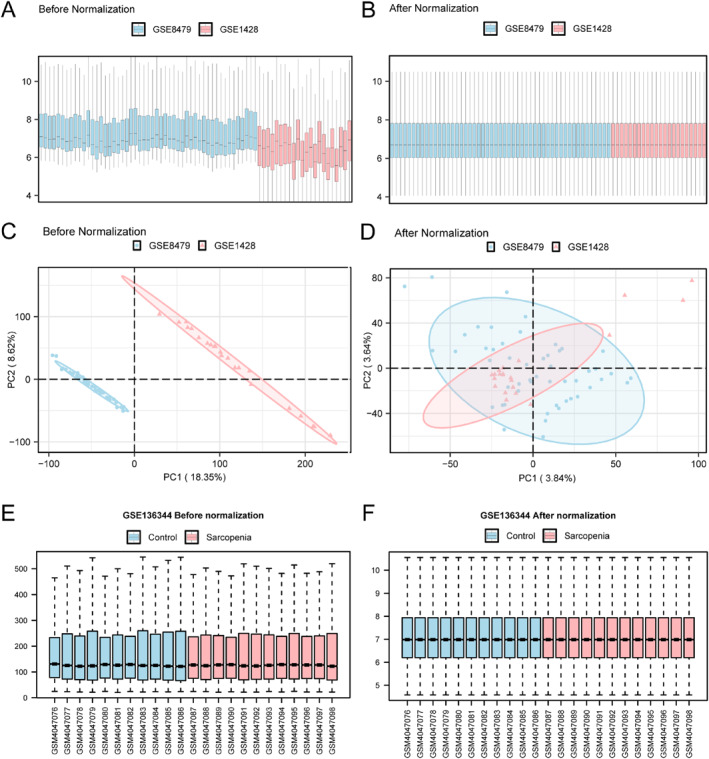
Assessment of batch effect correction and data standardisation. (A and B) Distribution boxplot showing pooled GEO datasets before (A) and following (B) batch correction. (C and D) PCA plot for the dataset before (C) and following (D) batch correction. (E and F) Distribution of gene expression in GSE136344 dataset before (E) and following (F) standardisation. PCA, Principal Component Analysis. Blue and pink in (E) represent the GSE8479 and GSE1428 datasets; whereas pink and blue in (F) represent the GSE136344 and control datasets separately.

R sva package (version 3.50.0) was employed for processing the GSE8479 and GSE1428 datasets for removing batch effects, and a pooled dataset was obtained. Differential expression before and following batch correction was analysed with distribution box plots (Figure 2A,B). PCA was then performed to compare low‐dimensional feature distributions prior to and after batch correction (Figure 2C,D). After normalisation, the expression distribution among samples became more concentrated and variability was markedly reduced, indicating the substantial elimination of batch effects in the sarcopenia datasets.

We categorised GSE136344 dataset in sarcopenia or control group. Following probe annotation and data standardisation, the expression distributions across samples were effectively normalised, as evidenced by the distribution box plots before and after this processing (Figure 2E,F).

### 3.3 Sarcopenia‐Related FAMRDEGs

**FIGURE 3:**
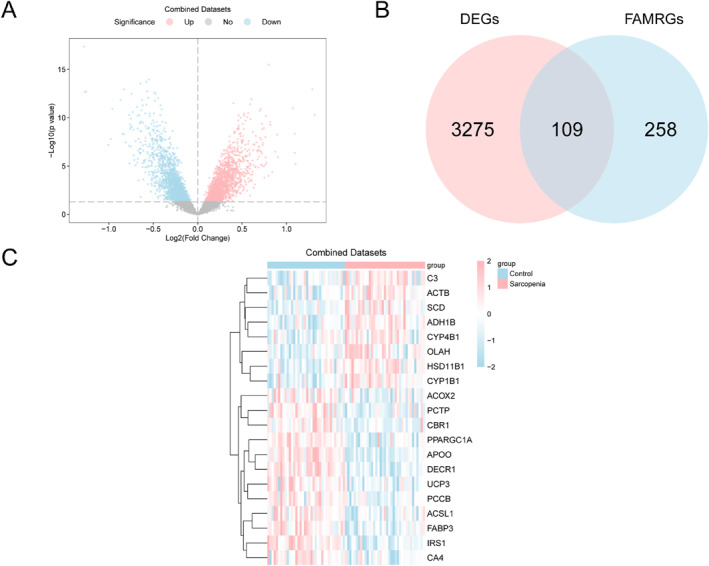
Differential analysis. (A) Volcano plot for DEGs. (B) Venn diagram for DEGs and FAMRGs. (C) Heatmap displaying top 20 FAMRDEGs (high expression in pink, low expression in blue). DEGs, Differentially Expressed Genes; FAMRGs, Fatty Acid Metabolism‐Related Genes.

There were altogether 3384 DEGs obtained, with 1707 showing up‐regulation whereas 1677 showing down‐regulation. A volcano plot of the DEGs was drawn based on these results (Figure 3A).

To obtain FAMRDEGs, the DEG‐FAMRG intersections were plotted as the Venn diagram (Figure 3B). There were altogether 109 FAMRDEGs identified, including *APOO*, *DECR1*, *IRS1*, *OPN3*, *IDH1*, *ALDH1B1*, *HPGD*, *HSD17B7*, *ECI2*, *PECR*, *ACOT13*, *PPARGC1A*, *HCCS*, *FABP3*, *CYP1B1*, *LGALS1*, *PTGES2*, *ADH1B*, *NDUFAB1*, *CS*, *ACTB*, *IGF1*, *ADRB2*, *SREBF2*, *CBR4*, *UCP3*, *PCCA*, *SCD*, *PCTP*, *ACSL1*, *C3*, *PCCB*, *FAAH*, *ETFA*, *TBXAS1*, *ABCD3*, *CBR1*, *OLAH*, *HSD11B1*, *CA4*, *SCARB2*, *HSD17B4*, *COG2*, *DECR2*, *DBI*, *SLC25A20*, *APOE*, *MAPKAPK2*, *ACADM*, *ABCC1*, *CYP4B1*, *PTGIS*, *ACAA2*, *LTC4S*, *ACOT8*, *PPARA*, *ACSL4*, *PHYH*, *SLC17A5*, *ACOX2*, *ALOX5AP*, *LEP*, *ACACA*, *PDK1*, *VEGFA*, *EDN1*, *PDK2*, *DHCR7*, *PRKAG2*, *HADHB*, *SLC2A4*, *MORC2*, *FADS3*, *SCP2*, *HPGDS*, *ACLY*, *PLA2G4A*, *ACAA1*, *LMNA*, *SLC25A17*, *ACBD4*, *MECR*, *PTGES3*, *PLIN1*, *HSD17B12*, *ABCD1*, *ALDH3A2*, *LPXN*, *SCAP*, *AKT1*, *AMACR*, *FABP5*, *RBP4*, *FAS*, *GCDH*, *FABP1*, *CD36*, *AGT*, *FASN*, *RXRA*, *APOA1*, *ALOX5*, *CYP11B1*, *CYP4F3*, *MLYCD*, *ICAM1*, *PTGS1*, *SLC27A6*, and *ADIPOR2*. A heatmap of the top 20 FAMRDEGs was generated (Figure 3C).

### 3.4 GO as Well as KEGG Analysis

**FIGURE 4:**
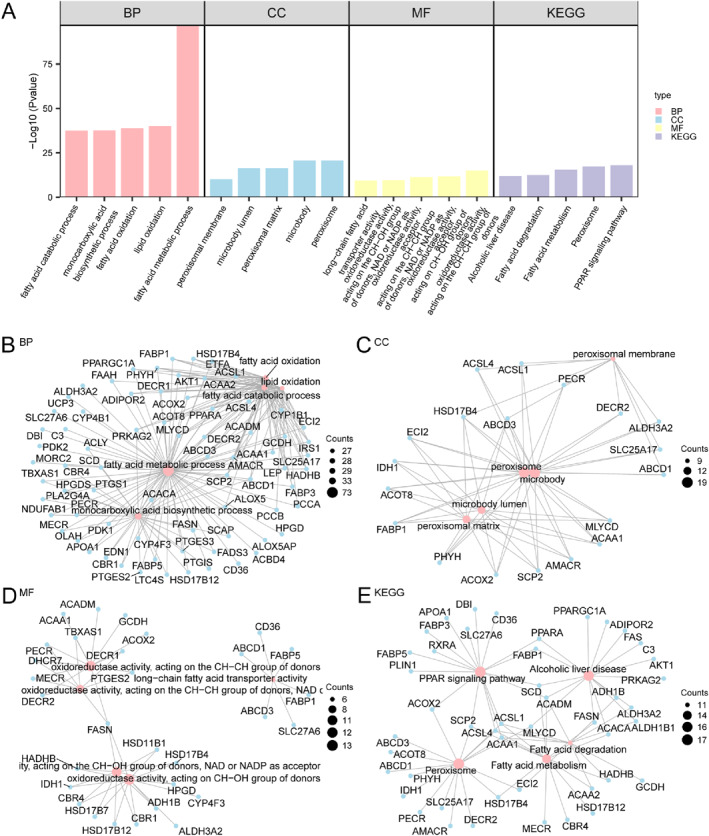
GO as well as KEGG analysis for FAMRDEGs. (A) Bar chart of the enriched GO and KEGG term. (B–E) Network diagrams for BP (B), CC (C), MF (D), and KEGG pathways (E). Pink nodes represent enriched terms, blue nodes stand for genes, whereas connecting lines indicate relations. Screening thresholds were *P* < 0.05 and FDR (*q* value) < 0.05, with BH used in *p* value correction.

GO alongside KEGG analysis was performed on 109 FAMRDEGs (Table 3). These genes were mostly associated with BPs like fatty acid oxidation, fatty acid metabolic process, lipid oxidation, monocarboxylic acid biosynthetic process, and fatty acid catabolic process; CCs such as peroxisome, microbody, peroxisomal matrix, microbody lumen, and peroxisomal membrane; and MFs such as oxidoreductase activity (acting on the CH–CH group of donors), oxidoreductase activity (acting on the CH–OH group of donors), and long‐chain fatty acid transporter activity. The identified genes exhibited significant enrichment in various biological pathways, such as PPAR pathway, peroxisome function, fatty acid degradation, fatty acid metabolism, and alcoholic liver disease, as illustrated in Figure 4A. Additionally, BP, CC, MF, and KEGG network diagrams were constructed (Figure 4B–E). For these diagrams, nodes of larger size represent more genes related to every respective entry.

**TABLE 3:** GO as well as KEGG enrichment on FAMRDEGs.

Ontology	ID	Description	GeneRatio	BgRatio	*p* value	p.adjust
BP	GO:0006631	Fatty acid metabolic process	73/109	395/18800	1.53231E‐97	4.07594E‐94
BP	GO:0034440	Lipid oxidation	29/109	112/18800	9.27511E‐41	1.23359E‐37
BP	GO:0019395	Fatty acid oxidation	28/109	106/18800	1.2826E‐39	1.13724E‐36
BP	GO:0072330	Monocarboxylic acid biosynthetic process	33/109	215/18800	2.77626E‐38	1.68995E‐35
BP	GO:0009062	Fatty acid catabolic process	27/109	102/18800	3.17659E‐38	1.68995E‐35
CC	GO:0005777	Peroxisome	19/109	141/19594	2.55434E‐21	2.69482E‐19
CC	GO:0042579	Microbody	19/109	141/19594	2.55434E‐21	2.69482E‐19
CC	GO:0005782	Peroxisomal matrix	12/109	50/19594	4.7965E‐17	2.53015E‐15
CC	GO:0031907	Microbody lumen	12/109	50/19594	4.7965E‐17	2.53015E‐15
CC	GO:0005778	Peroxisomal membrane	9/109	64/19594	7.78339E‐11	2.73716E‐09
MF	GO:0016627	Oxidoreductase activity, for donors in the CH–CH group	12/109	59/18410	8.8447E‐16	2.97182E‐13
MF	GO:0016614	Oxidoreductase activity, for donors in CH–OH group	13/109	140/18410	2.02696E‐12	3.4053E‐10
MF	GO:0016628	Oxidoreductase activity, for donors in CH–CH group, NAD or NADP as acceptor	8/109	29/18410	4.50305E‐12	5.04342E‐10
MF	GO:0016616	Oxidoreductase activity, for donors in the CH–OH group, NAD or NADP as acceptor	11/109	128/18410	2.55958E‐10	2.15005E‐08
MF	GO:0005324	Long‐chain fatty acid transporter activity	6/109	17/18410	4.39955E‐10	2.9565E‐08
KEGG	hsa03320	PPAR signalling pathway	17/92	76/8865	9.37926E‐19	2.11971E‐16
KEGG	hsa04146	Peroxisome	17/92	83/8865	4.72539E‐18	5.33969E‐16
KEGG	hsa01212	Fatty acid metabolism	14/92	57/8865	3.23179E‐16	2.43462E‐14
KEGG	hsa00071	Fatty acid degradation	11/92	43/8865	3.56849E‐13	2.0162E‐11
KEGG	hsa04936	Alcoholic liver disease	16/92	144/8865	1.11299E‐12	4.87036E‐11

Abbreviations: BP, Biological Process; CC, Cellular Component; FAMRDEGs, Fatty Acid Metabolism‐Related Differentially Expressed Genes; GO, Gene Ontology; KEGG, Kyoto Encyclopaedia of Genes and Genomes; MF, Molecular Function.

### 3.5 GSEA

**FIGURE 5:**
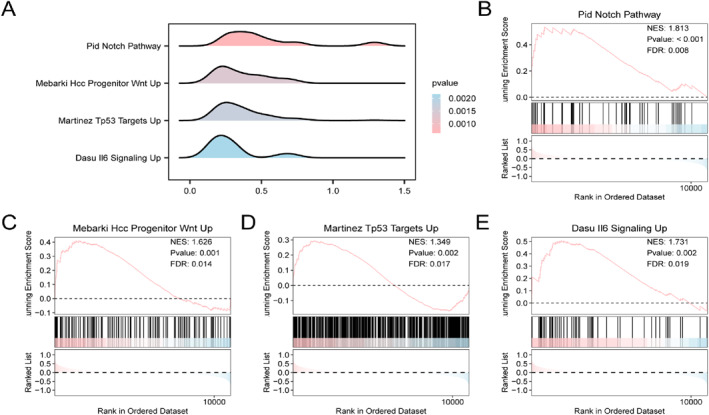
Differential analysis and GSEA on pooled datasets. (A) GSEA mountain plots for four enriched biological functions. (B–E) Enriched gene sets including Pid Notch Pathway (B), Mebarki Hcc Progenitor Wnt Up (C), Martinez Tp53 Targets Up (D), and Dasu Il6 Signalling Up (E). The colour gradient represents *p* values, where red indicates lower *p* values and blue indicates higher *p* values. Thresholds for GSEA were *P* < 0.05 and FDR (*q*‐value) < 0.05 using Benjamini–Hochberg correction.

To determine the relation of gene expression patterns with sarcopenia pathogenesis in integrated datasets, GSEA was performed utilising the log fold change (logFC) values for all genes within the sarcopenia and control cohorts, so as to evaluate the relation between gene expression profiles and the enriched BP, CC, as well as MF terms. The resulting enrichment landscape was illustrated in the form of a mountain plot (Figure 5A), with additional details provided in Table 4. Notably, the integrated datasets exhibited significant enrichment for various functional gene sets, including Mebarki Hcc Progenitor Wnt Up, Pid Notch Pathway, Dasu Il6 Signalling Up, and Martinez Tp53 Targets Up (Figure 5B–5E).

**TABLE 4:** GSEA results of pooled datasets.

ID	setSize	enrichmentScore	NES	*p* value	p.adjust	*q* value
NOTCH_PATHWAY	49	0.534841082	1.812953392	0.000636388	0.010117683	0.008235313
MEBARKI_HCC_PROGENITOR_WNT_UP	127	0.410046023	1.626028969	0.001380222	0.017667459	0.014380471
MARTINEZ_TP53_TARGETS_UP	496	0.294669716	1.348892408	0.001721361	0.020531851	0.01671195
DASU_IL6_SIGNALLING_UP	53	0.505956672	1.731320946	0.002162674	0.023917843	0.019467987

Abbreviation: GSEA, Gene Set Enrichment Analysis.

### 3.6 Sarcopenia Diagnostic Model Construction

**FIGURE 6:**
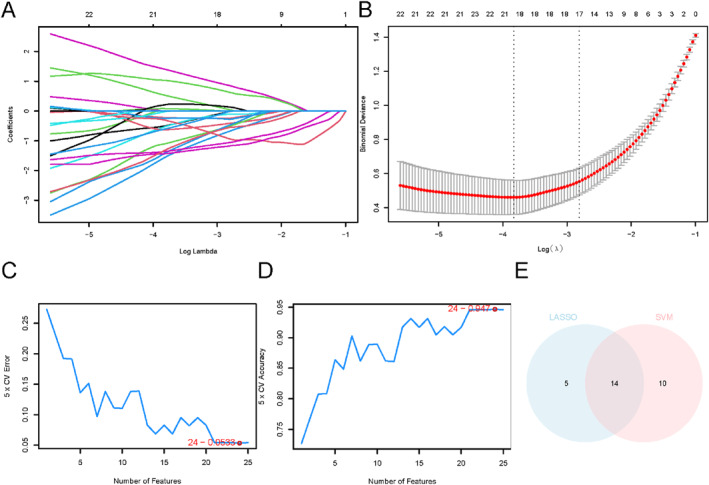
Sarcopenia diagnostic model establishment. (A) A diagram depicting variable loci of FAMRDEGs generated through LASSO regression within the pooled GEO dataset. (B) A diagram illustrating the regression model utilised in LASSO diagnostic framework. (C) The gene number obtained with SVM algorithm that exhibited the lowest error rate. (D) The gene number identified by SVM algorithm that demonstrated the highest accuracy. (E) The Venn diagram displaying the intersected gene set derived from LASSO and SVM algorithms. FAMRDEGs, Fatty Acid Metabolism‐Related Differentially Expressed Genes. LASSO, Least Absolute Shrinkage and Selection Operator. SVM, Support Vector Machine.

Among 103 FAMRDEGs significantly associated with sarcopenia within logistic regression model (*P* < 0.05; Table S2), we used LASSO regression for further establishing the diagnostic model. A LASSO coefficient trajectory plot (Figure 6A) alongside a LASSO model diagram (Figure 6B) was generated. Nineteen FAMRDEGs were selected as model genes in the LASSO regression, including *PCTP*, *FABP3*, *IGF1*, *PECR*, *ALDH1B1*, *SREBF2*, *IDH1*, *PPARGC1A*, *HPGD*, *APOO*, *ADH1B*, *ACOT8*, *DECR1*, *C3*, *FABP5*, *IRS1*, *LGALS1*, *OPN3*, and *HSD17B7*.

Subsequently, a SVM model was trained on the 103 FAMRDEGs for minimising error rate (Figure 6C) while maximising the predictive accuracy (Figure 6D). Notably, the SVM model achieved optimal performance with 24 genes, indicating minimal error and maximum predictive accuracy while maintaining a balance between feature information and noise. These 24 FAMRDEGs included *HPGD*, *IDH1*, *APOO*, *HSD17B7*, *OPN3*, *PECR*, *FABP3*, *SCD*, *DECR1*, *ACOT8*, *PTGES2*, *PPARGC1A*, *ACSL1*, *PCTP*, *ACOT13*, *ADH1B*, *HSD17B4*, *NDUFAB1*, *SREBF2*, *IRS1*, *CYP1B1*, *ACTB*, *MAPKAPK2*, *CA4*.

In order to find out key genes, FAMRDEGs obtained by LASSO regression model were intersected with those from SVM model. There were 14 FAMRDEGs acquired in later analysis (Figure 6E). The 14 FAMRDEGs were *PCTP*, *FABP3*, *PECR*, *SREBF2*, *IDH1*, *PPARGC1A*, *HPGD*, *APOO*, *ADH1B*, *ACOT8*, *DECR1*, *IRS1*, *OPN3*, and *HSD17B7*. This cross‐screening strategy effectively reduced algorithmic bias and enhanced the reliability of key gene screening.

### 3.7 Sarcopenia Diagnostic Model Verification

**FIGURE 7:**
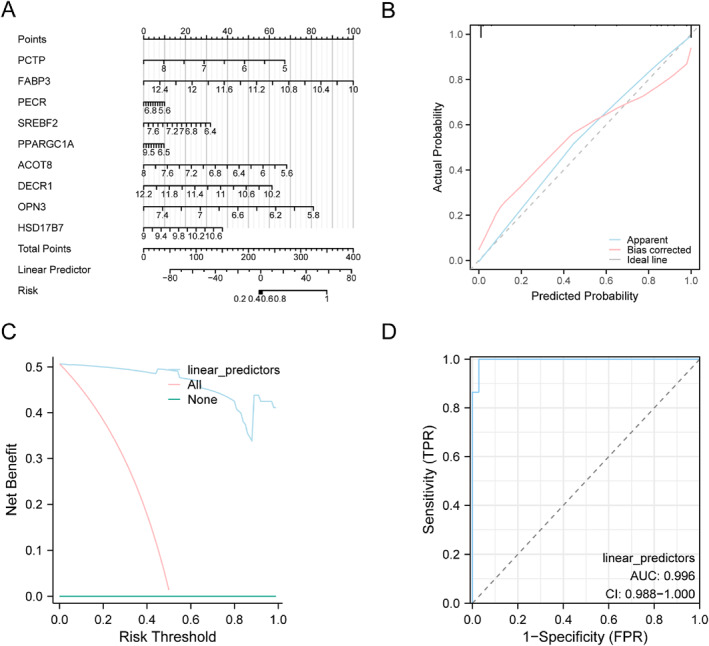
Sarcopenia diagnostic ability and validation. (A) Nomogram for key genes within a pooled dataset of GEO in a sarcopenia diagnostic model. (B and C) Sarcopenia diagnostic model was established on the basis of Calibration curve (B) and DCA curve (C) for key genes in the integrated GEO dataset. (D) Logistic regression model for the linear predictors used for ROC analysis in the integrated GEO dataset. In the DCA plot, horizontal and vertical coordinates suggest threshold probability and net return, separately. DCA, Decision Curve Analysis; ROC, Receiver Operating Characteristic; AUC, Area Under the Curve, with a value > 0.9 indicating high accuracy.

To further verify the diagnostic performance of the as‐prepared sarcopenia diagnostic model, we established nomograms by introducing 14 FAMRDEGs to demonstrate their correlations in the pooled datasets (Figure 7A). There were 9 model genes obtained from the multifactor regression model, namely, *PCTP*, *FABP3*, *PECR*, *SREBF2*, *PPARGC1A*, *ACOT8*, *DECR1*, *OPN3*, *and HSD17B7*, and they were key genes in later analysis. Among them, *FABP3* expression demonstrated better performance than the other parameters did in the sarcopenia diagnostic model.

Next, to analyse our constructed sarcopenia diagnostic model for its accuracy and resolution, we plotted the calibration curve on the basis of the calibration analysis. The model diagnostic performance for actual probabilities was subsequently assessed through fitting model‐predicted and measured probabilities upon different conditions (Figure 7B). For our diagnostic model, its calibration curve showed minimal deviation from the ideal diagonal (dashed line), indicating good agreement between predicted and observed probabilities.

We also used key genes from the pooled dataset for assessing the model's clinical utility through DCA. The findings are displayed in Figure 7C. Across a wide range, the model curve demonstrated a dominant position with a higher net benefit than both reference strategies, indicating its robust clinical value.

DCA of the key genes was performed to evaluate the model’s clinical utility. As shown in Figure 7C, this model demonstrated superior net benefits within various threshold probabilities, suggesting the candidate value for clinical decision‐making.

Moreover, an ROC curve for linear predictive factors in logistic regression model for diverse groups (sarcopenia/control) in the pooled datasets was drawn, and the results are presented (Figure 7D), revealing the favourable predictive significance of this logistic regression model for the pooled datasets.

### 3.8 PPI Network

**FIGURE 8:**
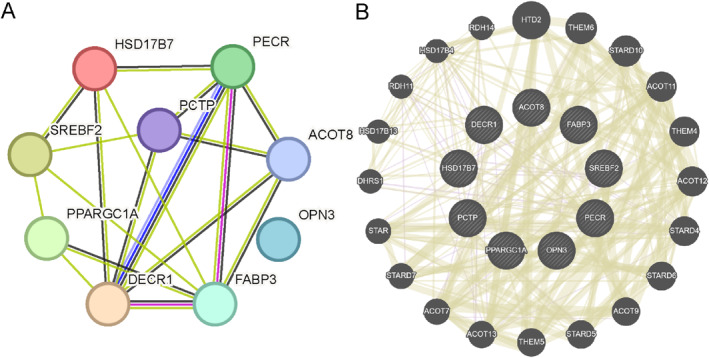
PPI network. (A) PPI network of key genes. (B) Predicted interaction network for functionally similar genes. Circular nodes stand for genes, whereas node size suggests gene attributes or importance. Edges represent molecular relations or functional interactions, and thicker lines indicates stronger associations.

Based on the STRING database, PPI analysis was conducted for the 9 key genes (*PCTP*, *FABP3*, *PECR*, *SREBF2*, *PPARGC1A*, *ACOT8*, *DECR1*, *OPN3*, *HSD17B7*), and a PPI network was constructed at a confidence score threshold > 0.150 (Figure 8A).

Next, genes functionally associated with 9 key genes were identified by GeneMANIA database, later, an extended interaction network illustrating physical interactions, co‐expression, gene co‐occurrence, and shared protein domains was constructed (Figure 8B). *FABP3*, *PPARGC1A*, and *SREBF2* received the highest relevance scores, indicating that these genes may serve as more prominent mediators linking sarcopenia with fatty acid metabolism.

### 3.9 Regulatory Network Establishment

**FIGURE 9:**
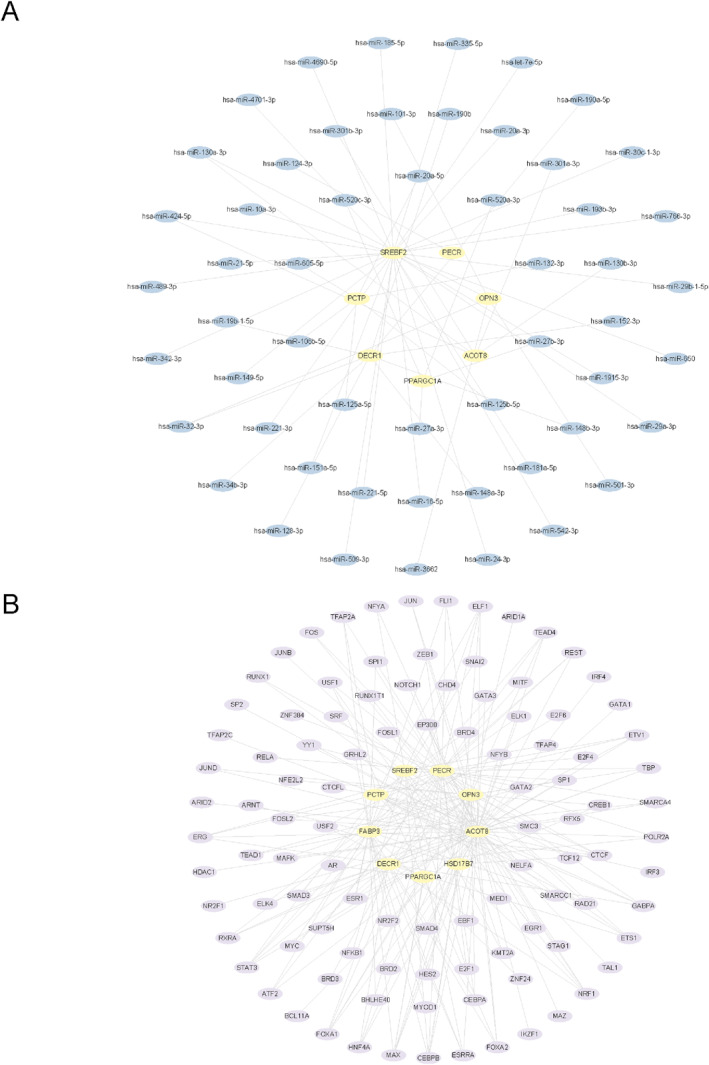
Regulatory network of key genes. (A) mRNA–miRNA regulatory network of key genes. (B) mRNA–TF regulatory network of key genes. Yellow nodes indicate mRNAs, blue indicate miRNAs, and purple indicate TFs.

Based on the key gene‐associated miRNAs obtained based on StarBase database, we built the mRNA‐miRNA network (Figure 9A). The network involved 7 key genes and 54 miRNAs (Table S3). Among them, miR‐130a‐3p, miR‐32‐3p, and miR‐424‐5p each targeted two key genes, suggesting that these miRNAs may exert important regulatory effects on FAM in sarcopenia.

Subsequently, TFs binding to the key genes were screened from the ChIPBase database, thereby enabling mRNA‐TF network construction (Figure 9B). In total, 9 key genes along with 102 TFs were involved (Table S4). Among these TFs, CEBPB regulated 6 key genes, whereas NRF1 and FOXA1 regulated 5 key genes each, indicating potential transcriptional regulatory mechanisms worthy of further investigation.

### 3.10 Differences in Key Gene Expression

**FIGURE 10:**
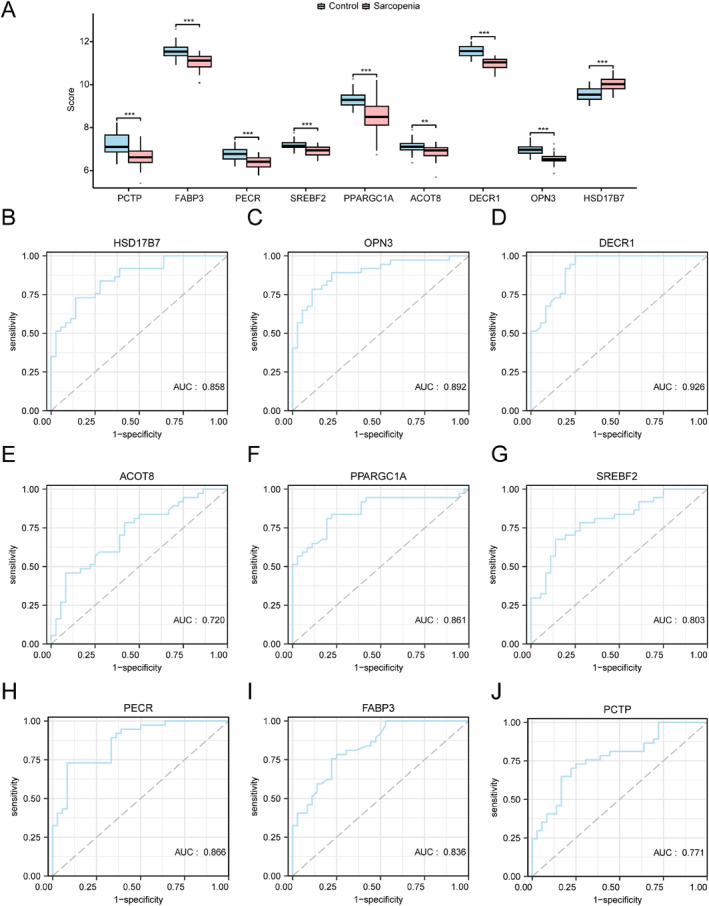
Differential expression, correlation, and ROC curve analyses. (A) Key gene levels were compared in sarcopenia compared with control groups in the pooled GEO datasets. (B–J) ROC curves for key genes *HSD17B7* (B), *OPN3* (C), *DECR1* (D), *ACOT8* (E), *PPARGC1A* (F), *SREBF2* (G), *PECR* (H), *FABP3* (I), and *PCTP* (J). **p < 0.01; ***p < 0.001.

To explore the 9 key genes for their differential expression in sarcopenia compared with control groups, group comparison plots were generated, as depicted in Figure 10A. *ACOT8* expression showed significant differences (*p* < 0.01), whereas *HSD17B7*, *OPN3*, *DECR1*, *PPARGC1A*, *SREBF2*, *PECR*, *FABP3*, and *PCTP* demonstrated highly significant differences (*p* < 0.001).

ROC curve for every key gene was drawn using pROC in R (version 1.18.5; Figure 10B–J). *HSD17B7*, *OPN3*, *ACOT8*, *PPARGC1A*, *SREBF2*, *PECR*, *FABP3*, and *PCTP* achieved moderate diagnostic ability (AUC 0.7–0.9), whereas *DECR1* exhibited high diagnostic accuracy (AUC > 0.9). Thus, *DECR1*, *HSD17B7* (AUC > 0.85), and *OPN3* (AUC > 0.85) may serve as strong diagnostic biomarkers for sarcopenia.

### 3.11 Correlation Analysis

**FIGURE 11:**
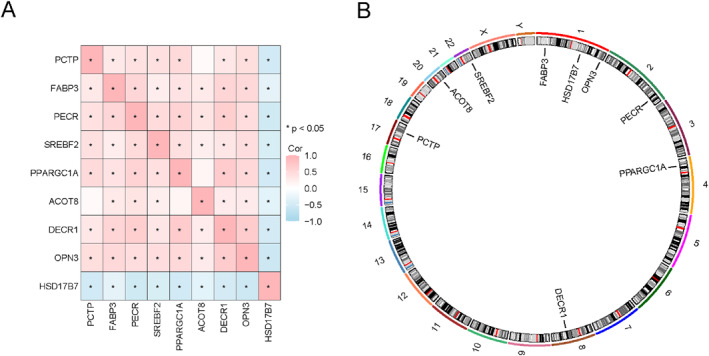
Correlation analysis on key genes and chromosome localisation. Correlation analysis (A) and chromosomal mapping (B) for key genes within human genome.

We constructed a correlation heatmap according to the 9 key gene expression matrix from pooled datasets (Figure 11A). *OPN3* and *DECR1*, as well as *DECR1* and *PPARGC1A*, showed positive correlations, whereas *PCTP* and *HSD17B7*, and *HSD17B7* and *PECR* exhibited negative correlations.

These 9 key genes were also investigated for their locations on chromosomes (Figure 11B). *FABP3*, *HSD17B7*, and *OPN3* were located on chromosome 1, whereas *PECR*, *PPARGC1A*, *DECR1*, *PCTP*, *ACOT8*, and *SREBF2* were located on chromosomes 2, 4, 8, 17, 20, and 22, respectively.

### 3.12 Grouping Comparison Diagram Analysis

**FIGURE 12:**
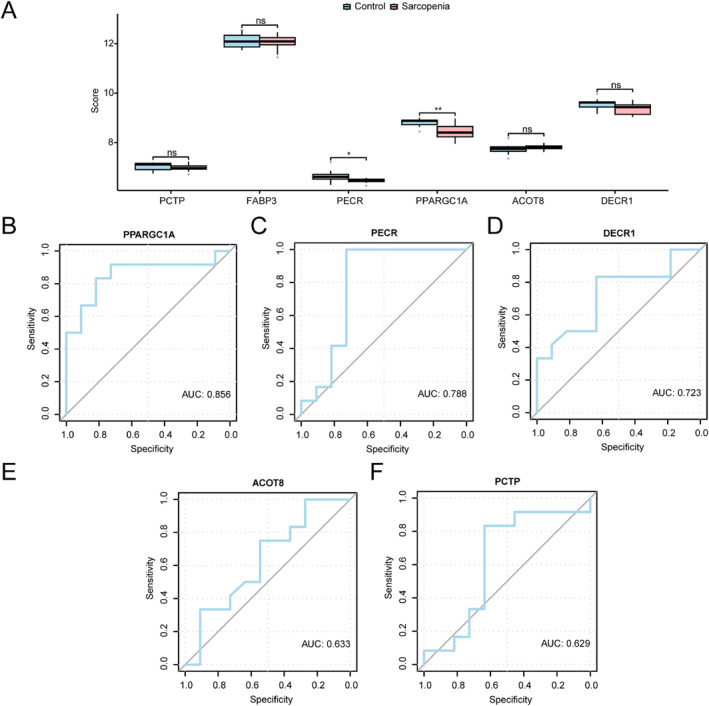
Differential analysis on key genes of different subgroups from GSE136344 dataset. (A) Comparison of key genes between sarcopenia group and control groups from GSE136344 dataset. (B–F) ROC curve of key genes *PPARGC1A* (B), *PECR* (C), *DECR1* (D), *ACOT8* (E), *PCTP* (F) from GSE136344 dataset. AUC, Area Under the Curve. * Indicates *p* < 0.05, ** represents *p* < 0.01.

The 9 key genes were analysed for their differential expression in sarcopenia versus control samples from GSE136344 dataset, a grouping comparison diagram was generated (Figure 12A). *PPARGC1A* was highly significantly different (*p* < 0.01), and *PECR* exhibited a significant difference (*p* < 0.05).

ROC curves were also generated for the 9 key genes in the GSE136344 dataset. Genes with AUC < 0.6 (*FABP3*, *SREBF2*, *OPN3*, *HSD17B7*) were excluded from display (Figure 12B–F). *PPARGC1A*, *PECR*, and *DECR1* demonstrated moderate diagnostic ability (AUC 0.7–0.9), whereas *ACOT8* and *PCTP* exhibited lower diagnostic ability (AUC 0.5–0.7).

### 3.13 Construction of High‐Low Rating Grouping of Fatty Acid Metabolism

**FIGURE 13:**
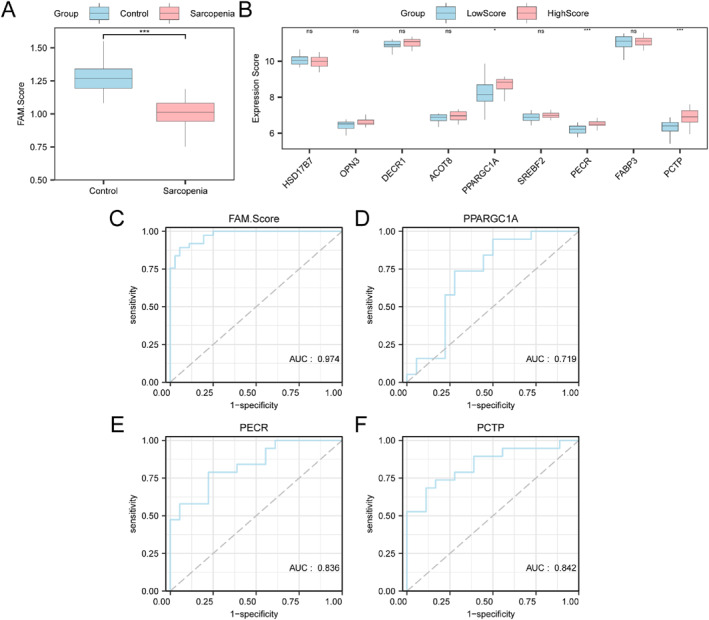
ssGSEA score analysis. (A) Subgroup comparison of fatty acid metabolism score (FAM‐Score) in sarcopenia versus control groups from pooled datasets. (B) Subgroup comparisons of key genes in HighScore versus LowScore groups of sarcopenia samples. (C) ROC curve for FAM‐Score from pooled datasets. (D–F) ROC curves for *PPARGC1A* (D), *PECR* (E), and *PCTP* (F) in sarcopenia samples. AUC, Area Under the Curve; FAM‐Score, Fatty Acid Metabolism Score; ROC, Receiver Operating Characteristic; ssGSEA, single‐sample Gene‐Set Enrichment Analysis. ns denotes *p* ≥ 0.05; *p < 0.05; ***p < 0.001. Pink and blue stand for sarcopenia/HighScore and control/LowScore groups separately.

According to key gene levels from pooled datasets, fatty acid metabolism scores (FAM‐Scores) were determined for all samples using ssGSEA algorithm. Subgroup comparison plots demonstrated that the FAM‐Score exhibited an obvious difference in sarcopenia versus control samples (*P* < 0.001; Figure 13A). Sarcopenia patients were then classified in HighScore or LowScore group based on median FAM‐Score (Figure 13B). *PPARGC1A* showed statistical significance (*p* < 0.05), whereas *PECR* and *PCTP* exhibited high significance (*p* < 0.001).

We subsequently drew ROC curves for FAM‐Score in pooled datasets (Figure 13C) and for the 3 key genes in sarcopenia samples (Figure 13D–F). The FAM‐Score exhibited high diagnostic ability (AUC > 0.9), whereas *PPARGC1A*, *PECR*, and *PCTP* showed moderate diagnostic performance for distinguishing HighScore and LowScore groups (0.7 < AUC < 0.9).

### 3.14 Immune Infiltration Analysis

**FIGURE 14:**
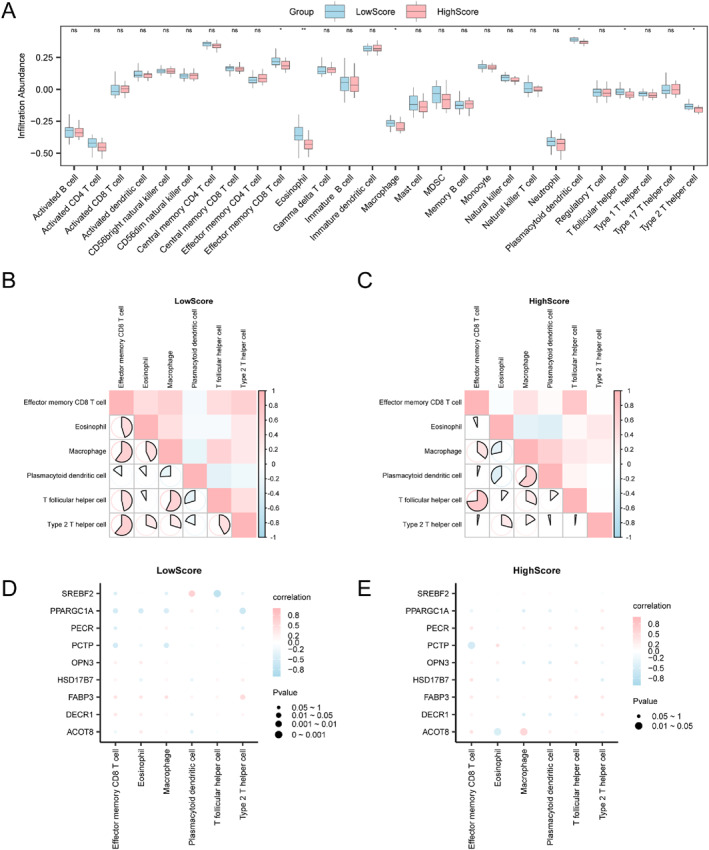
Immune infiltration analysis using ssGSEA algorithm. (A) Subgroup comparison of immune cells from sarcopenia samples showing a LowScore for FAM versus a HighScore for FAM. (B and C) FAM (LowScore) group (B) and FAM in sarcopenia samples correlation results for immune cell infiltration abundances of sarcopenia (HighScore) group (C). FAM (LowScore) group (D) and FAM in sarcopenia samples bubble map showing correlation of immune cell infiltration abundances with key genes from the HighScore (E) group of sarcopenia. Blue is the LowScore group of Fatty Acid Metabolism, and pink is the HighScore group of Fatty Acid Metabolism. Pink and blue suggest positive and negative correlations, and colour depth suggests correlation strength. ssGSEA, single‐sample Gene‐Set Enrichment Analysis; ns represented not‐significant (*p* value ≥ 0.05). *p < 0.05; **p < 0.01.

We quantified the infiltration levels of 28 immune cell types in sarcopenia samples by applying the ssGSEA algorithm to the integrated expression matrix. Comparative analysis revealed a distinct pattern of differential infiltration across groups (Figure 14A). Specifically, six immune cell subtypes, namely plasmacytoid dendritic cells, eosinophils, effector memory CD8 T cells, macrophages, T follicular helper cells, and type 2 T helper cells, were significantly different (*p* < 0.05). Subsequently, correlation heatmaps (Figure 14B,C) were generated to present correlation analysis concerning immune cell abundances during immunoinfiltration analysis of sarcopenia samples.

Many immune cells demonstrated strong correlations within the low‐FAM score group of sarcopenia samples. Specifically, effector memory CD8 T cells and type 2 T helper cells exhibited most robust positive relations (*R* = 0.61, *P* < 0.05; Figure 14B). Notably, this association was even stronger in high‐score group (*R* = 0.735, *P* < 0.05), which exhibited broadly enhanced positive correlations across multiple immune cell types (Figure 14C), whereas follicular memory CD8 T cells also showed a similarly strong positive relation (*R* = 0.735, *P* < 0.05). Lastly, the correlation bubble map was employed for depicting key gene‐immune cell infiltration abundance relationships (Figure 14D,E). Our correlation bubble map indicated that numerous immune cells within sarcopenia samples characterised by low FAMs were strongly correlated, with SREBF2 being most significantly and inversely related to T helper cells (*R* = −0.746, *P* < 0.05) (Figure 14D). Most immune cells were similarly strongly correlated in high FAM‐score group, with ACOT8 being most significantly positively related to macrophages (*R* = 0.556, *P* < 0.05) (Figure 14E).

### 3.15 Sarcopenia Subtype Construction

**FIGURE 15:**
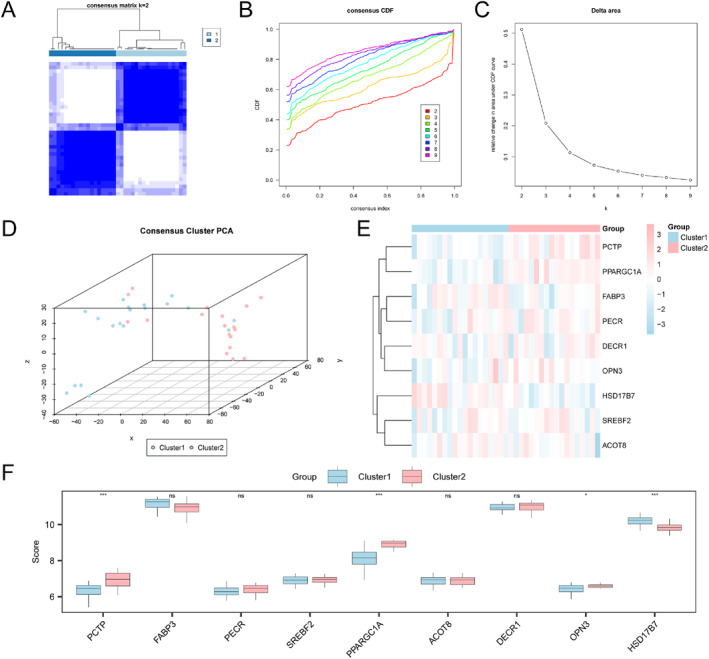
Consensus clustering of sarcopenia. (A) Consensus clustering of pooled datasets. (B and C) Empirical Cumulative Distribution Function (CDF) plot (B) and Delta plot (C). (D) 3D t‐SNE cluster plots showing two sarcopenia subtypes. (E) Heatmap for key gene levels in sarcopenia subtypes. (F) Key genes in both subtypes compared between groups. Blue and pink represent subtype A (Cluster1) and subtype B (Cluster2) separately. ns stand for not‐significant (*p* ≥ 0.05); *p < 0.05; ***p < 0.001.

To identify sarcopenia subtypes in the pooled datasets, ConsensusClusterPlus in R (version 1.72.0) was applied according to 9 key gene levels. There were two different sarcopenia subtypes obtained, designated as subtype A (Cluster 1, *n* = 19) as well as subtype B (Cluster 2, *n* = 18; Figure 15A–C). The 3D t‐SNE plots confirmed clear separation between the two subtypes (Figure 15D).

A heatmap generated using R‐pheatmap illustrated the key DEGs between both subtypes (Figure 15E). Group comparison analysis further confirmed that *OPN3* was significantly differentially expressed in two subtypes (*P* < 0.05), whereas *HSD17B7*, *PCTP*, and *PPARGC1A* showed highly significant differences (*p* < 0.001; Figure 15F).

### 3.16 Differences in Immune Cell Infiltration Levels

**FIGURE 16:**
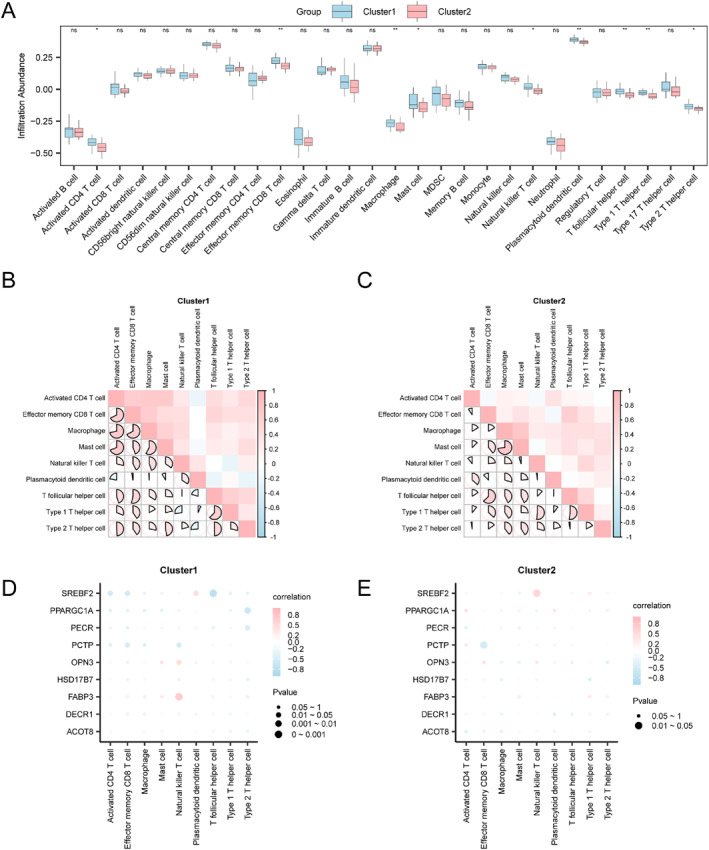
Immune infiltration landscape in sarcopenia risk clusters. (A) Immune cell infiltration levels compared between Cluster 1 and Cluster 2. (B and C) Heatmaps depicting intercellular correlations among immune infiltration levels within Cluster 1 (B) and Cluster 2 (C). (D and E) Bubble plots showing correlations of key gene expression with immune cell infiltration in Cluster 1 (D) and Cluster 2 (E). ssGSEA, single‐sample Gene‐Set Enrichment Analysis. Blue represents Cluster1 and pink represents Cluster2. Pink and blue squares suggest positive and negative correlations, and deeper colour reflects more potent correlation. ns denotes *p* ≥ 0.05; *p < 0.05; **p < 0.01.

Immune infiltration levels of 28 immune cell types in sarcopenia samples were calculated using the ssGSEA algorithm. Group comparison plots (Figure 16A) revealed that 9 immune cell types showed statistically significant differences (*p* < 0.05), such as effector memory CD8^+^ T cells, mast cells, T follicular helper cells, activated CD4^+^ T cells, macrophages, natural killer T cells, plasmacytoid dendritic cells, type 1 T helper cells, alongside type 2 T helper cells.

According to the correlation heatmaps (Figure 16B,C), the majority of immune cells within Cluster1 had positive associations, among which, macrophages and activated CD4^+^ T cells exhibited strongest associations (*R* = 0.719, *P* < 0.05). In Cluster2, numerous immune cells also displayed positive associations, and macrophages and mast cells had most potent correlations (*R* = 0.73, *P* < 0.05).

Correlation bubble maps (Figure 16D,E) illustrated the key gene‐immune infiltration level associations. In Cluster1, *SREBF2* was most negatively related to T helper cells (*R* = −0.772, *P* < 0.05). In Cluster2, *SREBF2* was most positively related to natural killer T cells (*R* = 0.583, *P* < 0.05). These findings suggest that key FAM‐related genes such as *SREBF2* may modulate immune cell infiltration through mechanisms involving immunometabolic reprogramming, oxidative stress, or energy metabolism.

### 3.17 Experimental Validation

**FIGURE 17:**
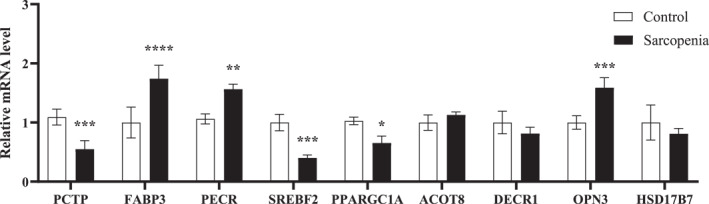
Validation of mRNA expression levels of 9 key genes. mRNA expression levels of *PCTP*, *FABP3*, *PECR*, *SREBF2*, *PPARGC1A*, *ACOT8*, *DECR1*, *OPN3*, and *HSD17B7* in C2C12 myoblasts obtained through qPCR (*n* = 3). *p < 0.05; **p < 0.01; ***p < 0.001.

The qPCR results demonstrated that *FABP3* (*P* < 0.001), *PECR* (*P* < 0.01), and *OPN3* (*P* < 0.001) expression markedly increased in D‐gal‐induced sarcopenia compared with control groups, whereas *PCTP* (*P* < 0.001), *SREBF2* (*P* < 0.001), and *PPARGC1A* (*P* < 0.05) levels dramatically decreased. *ACOT8* showed an upward trend, whereas *DECR1* and *HSD17B7* showed downward trends, although these changes were not statistically significant (Figure 17). These findings were consistent with the predictions derived from the bioinformatics analysis.

## 4 Discussion

Epidemiological data show that the sarcopenia prevalence among Chinese individuals aged ≥ 60 years is about 5.7%–3.9%, and increases markedly with age [35, 36], underscoring the urgent need to elucidate its mechanisms and develop therapeutic strategies. With advances in metabolomics and multi‐omics technologies, the molecular mechanisms of sarcopenia have gradually been explored. However, its complex pathophysiological network has not been fully resolved. Therefore, identifying differentially expressed genes associated with sarcopenia is crucial for discovering reliable diagnostic biomarkers.

In the present investigation, altogether 109 FAMRDEGs associated with sarcopenia were obtained. As revealed by GO as well as KEGG analysis, they were mostly clustered within BPs and pathways pertinent to fatty acid metabolism. Specifically, they were associated with processes such as fatty acid catabolism, lipid oxidation, fatty acid metabolism, fatty acid oxidation, monocarboxylic acid biosynthesis, fatty acid degradation, and peroxisome proliferator‐activated receptor (PPAR) pathway. To identify potential diagnostic markers, we utilised the LASSO and SVM models and identified 14 pivotal genes.

The LASSO regression model is particularly suitable for feature selection in high‐dimensional datasets by introducing an L1 penalty term (*λ*|*β*|), which compresses coefficients of less relevant variables to zero, thereby preventing overfitting and improving model generalisability. Meanwhile, the SVM model maintains robust classification performance under high noise or sample imbalance by constructing an optimal hyperplane to maximise the inter‐class margin. Cross‐screening genes from both models mitigated algorithmic bias and enhanced reliability, reproducibility, and biological interpretability.

A total of 9 key genes (*PCTP*, *FABP3*, *PECR*, *SREBF2*, *PPARGC1A*, *ACOT8*, *DECR1*, *OPN3*, and *HSD17B7*) were further supported through correlation and nomogram analyses. PPI and regulatory networks revealed their biological associations. According to ROC curve results, the above genes may be the candidate diagnostic biomarkers with AUC values ranging from 0.7 to 0.9. FAM‐Score analysis confirmed that three key genes differed significantly between HighScore and LowScore groups, with *PPARGC1A* showing statistical significance (*p* < 0.05) and *PECR* and *PCTP* showing strong significance (*p* < 0.001).

*PPARGC1A*, also known as PGC‐1α, is a key metabolism‐related transcriptional co‐activator widely distributed in organs such as the heart, liver, kidney, adipose tissue, brain, pancreas, and skeletal muscle. In the liver, glucagon and catecholamines induce PGC‐1α expression, which interacts with HNF‐4α, PPAR‐α, and FOXO1. In skeletal muscle, PGC‐1α expression can be triggered by cold stimulation and catecholamines. PGC‐1α co‐activates PPARs and promotes uncoupling protein expression to enhance adaptive thermogenesis. Exercise is an important stimulator of *PPARGC1A* expression in skeletal muscle, and PGC‐1α facilitates oxidative phosphorylation, mitochondrial biogenesis, and oxidative type I muscle fibre formation. In addition, PGC‐1α enhances the transcriptional activity of genes encoding fatty acid oxidases through co‐activation with PPAR‐α, thereby promoting fatty acid oxidative catabolism in skeletal muscle [37]. In our qPCR assays, *PPARGC1A* was down‐regulated in the sarcopenia versus control groups (*P* < 0.05). The above results conform to prior reports, underscoring the significance of *PPARGC1A* in sarcopenia.

*PCTP* regulates fatty acid synthesis and metabolism by interacting with PPARδ (peroxisome proliferator‐activated receptor δ). *PCTP* directly binds to the ligand‐binding domain (LBD) of PPARδ, thereby inhibiting its transcriptional activity. When *PCTP* is down‐regulated, PPARδ transcriptional activity is markedly enhanced, indicating that under physiological conditions *PCTP* acts as a negative regulator of PPARδ [38]. Because PPARδ exerts an important effect on regulating fatty acid oxidation and metabolism, its inhibition may increase fatty acid synthesis. In vascular smooth muscle cells, activation of PPARδ suppresses angiotensin II‐induced cellular senescence [39]. In our model, *PCTP* expression was significantly reduced in D‐gal‐induced C2C12 myoblasts (*P* < 0.001). This down‐regulation may enhance PPARδ activity by lifting transcriptional repression, thereby increasing fatty acid synthesis and aggravating metabolic dysregulation and senescence in muscle cells.

The PPAR family sustains skeletal muscle homoeostasis by regulating fatty acid oxidation, inflammatory signalling, and lipid metabolism. The enrichment of the PPAR signalling pathway in this study suggests that dysregulation of this pathway may exacerbate sarcopenia through multiple mechanisms: insufficient activation of PPARα/δ suppresses fatty acid oxidation genes, causing lipid accumulation and impaired energy production, whereas excessive activation of PPARγ promotes adipocyte differentiation and intermuscular fat infiltration, contributing to muscle atrophy [40]. Moreover, the PPAR pathway interacts with the mTOR signalling network. BCAA metabolic disruption can induce insulin resistance and autophagy inhibition through mTOR overactivation [41], whereas PPAR agonists have been shown to alleviate metabolic imbalances by modulating mTOR activity [42].

Six immune cell types—eosinophils, effector memory CD8 T cells, macrophages, type 2 T helper cells, plasmacytoid dendritic cells, and T follicular helper cells—showed significant differences and correlated with the FAM‐Score. The M1‐M2 spatiotemporal balance of macrophages exerts an essential effect on modulating inflammatory microenvironment during severe muscle injury [43, 44]. Perturbation of macrophage polarisation intensifies inflammation and promotes fibrosis, thereby compromising skeletal muscle regenerative capacity and increasing the risk of secondary injury [45]. Zhou et al. [46] reported that exosomal miR‐501 derived from M2 macrophages promoted myotube formation following injury, reinforcing the importance of macrophage polarisation in tissue repair.

In Cluster 1, pro‐inflammatory infiltration was prominent, with increased macrophages and activated CD4 T cells. Upregulated TNF‐α and IL‐6 suppress CPT1 and PPARγ expression, impairing fatty acid β‐oxidation and reducing energy supply to muscle fibres. In contrast, Cluster 2 exhibited macrophages and mast cells with a comparatively lower inflammatory burden. This subtype displayed only mild energetic defects, with slow disease progression and a milder clinical phenotype. Consequently, patient stratification based on clinical phenotypes (e.g., sex, age, muscle strength, endurance) may enable targeted interventions, such as fatty acid metabolism‐oriented nutritional support, high‐protein diets, and resistance training.

D‐gal is widely used as an inducer of age‐related phenotypes and fibrotic injury across tissues, including skeletal muscle [34]. Accordingly, it was used to establish a cell‐based model of sarcopenia in C2C12 myoblasts. qPCR assays confirmed consistent changes in *PCTP*, *SREBF2*, and *PPARGC1A* expression compared with bioinformatics predictions. *FABP3* levels increased significantly, *DECR1* showed a downward trend, *PECR* and *OPN3* were significantly upregulated, whereas *HSD17B7* exhibited a decreasing trend.

Although this study provides mechanistic insights into FAM dysregulation in sarcopenia, several limitations remain. First, the bioinformatics analysis relied on public GEO datasets, which may be limited by sample size or demographic representation, potentially affecting generalisability. Despite normalisation and batch correction, residual heterogeneity may persist. Second, the present analysis focused solely on transcriptional regulation and did not address post‐transcriptional or post‐translational mechanisms, which may also contribute to disease pathology. Future research incorporating proteomics or phosphoproteomics would provide a more comprehensive mechanistic understanding. Third, only qPCR was used for experimental validation. Additional experimental modalities and clinical tissue samples from human subjects are needed to confirm the stability and translatability of these biomarkers.

## 5 Conclusion

This study systematically mined public datasets to identify candidate biomarkers and established a preliminary risk assessment framework. Experimental validation using C2C12 myoblasts confirmed differential expression patterns of key genes, supporting the bioinformatics findings. This integrated strategy improves understanding of the molecular mechanisms underlying sarcopenia. Future interdisciplinary approaches are anticipated to further elucidate the mechanistic landscape and accelerate translational application.

## Author Contributions

**Ruopeng Yang:** writing – original draft. **Shan Gu:** writing – original draft. **Yang Li:** funding acquisition, writing – review and editing. **Ping Xia:** supervision, writing – review and editing.

## Funding

This work was supported by the Key Project of Scientific Research of Traditional Chinese Medicine of Hubei Administration of Traditional Chinese Medicine (Grant ZY2025D007), the Science and Technology Programme of Hubei Province (Grant 2024BCB035), Centralised Guided Local Science and Technology Development Funding Project (Grants 2024EIA037, 2024EIA038), and Hubei Provincial Natural Science Foundation Joint Fund Project (Grant 2024AFD332, 2025AFD519, 2022CFD149).

## Conflicts of Interest

The authors declare no conflicts of interest.

## Supporting information


**Table S1:** The information of 367 FAMRGs.

**Table S2:** The information of FAMRDEGs.

**Table S3:** The network relations of 7 key genes and 54 miRNAs.

**Table S4:** The network relations of 9 key genes and 102 TFs.

## Data Availability

The data that support the findings of this study are available upon request from the corresponding author.
