# Supplementary material for: Exploring and Validating the Molecular Mechanisms Linking Fatty Acid Metabolism and Sarcopenia
Source: IET Syst Biol. 2025 Dec 29;20(1):e70052. doi: 10.1049/syb2.70052 (PMC12747248; doi:10.1049/syb2.70052)
Supplement: Supplementary file 1 — Table S1: The information of 367 FAMRGs. [file SYB2-20-e70052-s004.docx]

Table S1. The information of 367 FAMRGs

| FAMRGs |
| --- |
| \| FADS1 \| \| --- \| \| ACADM \| \| APOE \| \| LDLR \| \| LIPC \| \| FASN \| \| ACADVL \| \| ABCG5 \| \| CETP \| \| ACACA \| \| ABCG8 \| \| CPT1A \| \| CPT2 \| \| ACADL \| \| SCD \| \| FADS2 \| \| ACSL6 \| \| INS \| \| ACACB \| \| CD36 \| \| ACOX1 \| \| PPARA \| \| ACSM2A \| \| AMACR \| \| ACSM3 \| \| ABCD1 \| \| LACC1 \| \| ACSM6 \| \| PPARGC1A \| \| LPL \| \| PPARG \| \| ACSL4 \| \| EHHADH \| \| GGT1 \| \| PPARD \| \| SCP2 \| \| ACSL5 \| \| ACSF2 \| \| LIPE \| \| PRKAA2 \| \| SLC27A2 \| \| CPT1B \| \| SREBF1 \| \| ADIPOQ \| \| PON1 \| \| PHYH \| \| THEM4 \| \| THEM5 \| \| DBI \| \| CROT \| \| ELOVL7 \| \| LEP \| \| PCSK9 \| \| ACADS \| \| PDK4 \| \| CYP4A11 \| \| ACBD5 \| \| SIRT1 \| \| NR1H2 \| \| UCP1 \| \| SLC25A20 \| \| ACOT8 \| \| CYP4F2 \| \| CYP4F3 \| \| HADHA \| \| HSD17B4 \| \| FABP2 \| \| FABP4 \| \| HNF4A \| \| CYP1A1 \| \| CYP2C19 \| \| ACOT7 \| \| ACOT2 \| \| HADH \| \| ECI2 \| \| ALB \| \| ECHS1 \| \| ACOXL \| \| HPGD \| \| ACOT11 \| \| GHRL \| \| MLYCD \| \| RXRA \| \| ALOX15 \| \| PTGIS \| \| SLC25A17 \| \| SLC22A5 \| \| ACLY \| \| ELOVL6 \| \| TBXAS1 \| \| HACL1 \| \| ACOT12 \| \| HAO2 \| \| ACOT1 \| \| CYP4F8 \| \| ACOT6 \| \| TCF7L2 \| \| HADHB \| \| ACSM1 \| \| CBS \| \| CYP3A4 \| \| ACSM4 \| \| CYP4V2 \| \| RETN \| \| DVL2 \| \| ACSM5 \| \| PLA2G4A \| \| APOC3 \| \| CNR1 \| \| GPT \| \| ACSM2B \| \| ACSL1 \| \| ABCG1 \| \| ACSL3 \| \| MMAA \| \| TNF \| \| DLG4 \| \| LTC4S \| \| MCEE \| \| ACSBG1 \| \| ACTB \| \| MB \| \| SLC17A5 \| \| STAR \| \| APOC2 \| \| PTGS2 \| \| EPHX2 \| \| ELOVL5 \| \| ALOX15B \| \| PRKAG2 \| \| CYP2U1 \| \| ACAA2 \| \| CYP2J2 \| \| SLC27A3 \| \| ACAD11 \| \| ALOX5AP \| \| ELOVL4 \| \| ACSF3 \| \| SCD5 \| \| FABP5 \| \| UCP2 \| \| ALOX5 \| \| CYP2C8 \| \| CYP2C9 \| \| CRAT \| \| CYP1A2 \| \| ACOX2 \| \| ACAD10 \| \| ACOT13 \| \| ACSBG2 \| \| CYP4A22 \| \| PTGS1 \| \| SLC25A1 \| \| MCAT \| \| PPT2 \| \| ECI1 \| \| ELOVL2 \| \| ACOT4 \| \| ELOVL3 \| \| UCP3 \| \| CYP2E1 \| \| FAS \| \| SREBF2 \| \| SLC27A1 \| \| ABCC1 \| \| CYP1B1 \| \| FAAH \| \| GPX4 \| \| PPT1 \| \| ALDH3A2 \| \| PCCA \| \| PCCB \| \| AKR1C3 \| \| HSD17B3 \| \| MAPKAPK2 \| \| MMUT \| \| PRKAB2 \| \| PTGDS \| \| ALOX12 \| \| LTA4H \| \| CBR1 \| \| DPEP1 \| \| PON3 \| \| PTGES3 \| \| GPX1 \| \| MECR \| \| PON2 \| \| ALOX12B \| \| ALOXE3 \| \| PTGES \| \| PTGES2 \| \| ACAA1 \| \| CYP4B1 \| \| ELOVL1 \| \| GPX2 \| \| NDUFAB1 \| \| TECR \| \| ACOX3 \| \| MORC2 \| \| CYP8B1 \| \| DECR1 \| \| DPEP2 \| \| GGT5 \| \| HACD1 \| \| HSD17B12 \| \| MID1IP1 \| \| HPGDS \| \| ACBD4 \| \| CYP4F11 \| \| CYP4F22 \| \| HSD17B8 \| \| PCTP \| \| PECR \| \| PTGR2 \| \| ACOT9 \| \| CBR4 \| \| DECR2 \| \| NUDT7 \| \| PTGR1 \| \| TECRL \| \| HACD3 \| \| NUDT19 \| \| OLAH \| \| ACBD6 \| \| FAAH2 \| \| HACD2 \| \| THRSP \| \| ACBD7 \| \| DPEP3 \| \| HACD4 \| \| PRXL2B \| \| AWAT1 \| \| HTD2 \| \| IL6 \| \| HIF1A \| \| APOO \| \| SLC2A4 \| \| SLC25A10 \| \| IRS1 \| \| SERPINE1 \| \| IL10 \| \| SCARB1 \| \| APOB \| \| PNPLA2 \| \| ADRB3 \| \| ADIPOR2 \| \| FABP3 \| \| INSR \| \| APOA1 \| \| CCL2 \| \| IGF1 \| \| ABCA1 \| \| HMGCR \| \| IL1B \| \| VCAM1 \| \| MTTP \| \| GPR84 \| \| LPIN3 \| \| PRKAA1 \| \| LEPR \| \| EDN1 \| \| LIPA \| \| NR1H4 \| \| POMC \| \| FABP12 \| \| NPY \| \| FABP1 \| \| ACE \| \| TLR4 \| \| HSD11B1 \| \| ICAM1 \| \| AGT \| \| NOS3 \| \| ADRB2 \| \| ANGPTL3 \| \| CRP \| \| SCARB2 \| \| APOA2 \| \| SELE \| \| MLXIPL \| \| CYP7A1 \| \| LIPG \| \| SCAP \| \| SHBG \| \| GCDH \| \| AKT1 \| \| C3 \| \| RBP4 \| \| SI \| \| G6PC1 \| \| SOAT1 \| \| SOAT2 \| \| GCG \| \| FGF21 \| \| PDK1 \| \| PDK2 \| \| OPN3 \| \| ALKBH7 \| \| PIK3C3 \| \| VEGFA \| \| SLC27A4 \| \| DGAT1 \| \| CDY1 \| \| CDY1B \| \| DPP4 \| \| LMNA \| \| PLA2G7 \| \| PTPN1 \| \| REN \| \| DHCR7 \| \| LCAT \| \| GLP1R \| \| SLC5A2 \| \| CYP11B1 \| \| PDE5A \| \| NPC1L1 \| \| PLTP \| \| CYP21A2 \| \| FOXC2 \| \| MGAM \| \| USF1 \| \| APOA5 \| \| LPA \| \| TAFAZZIN \| \| XPR1 \| \| LDLRAP1 \| \| COG2 \| \| LPXN \| \| RETNLB \| \| PDP1 \| \| FADS3 \| \| CD46 \| \| ABCD3 \| \| PRKAB1 \| \| KLHL25 \| \| ETFA \| \| CS \| \| PEX14 \| \| ABCD4 \| \| ACAD9 \| \| PLIN1 \| \| SLC27A6 \| \| CEL \| \| CA4 \| \| CPOX \| \| HSD17B11 \| \| HSPH1 \| \| ALDH1B1 \| \| DHCR24 \| \| HCCS \| \| TDO2 \| \| CA2 \| \| AOC3 \| \| ACAT1 \| \| GABARAPL1 \| \| NSDHL \| \| ACAT2 \| \| H2AZ1 \| \| ALDOA \| \| SERINC1 \| \| INMT \| \| ADH1B \| \| LGALS1 \| \| HSP90AA1 \| \| GPD2 \| \| IDH1 \| \| HSD17B7 \| |

FAMRGs: fatty acid metabolism related genes.
