# Supplementary material for: Exploring and Validating the Molecular Mechanisms Linking Fatty Acid Metabolism and Sarcopenia
Source: IET Syst Biol. 2025 Dec 29;20(1):e70052. doi: 10.1049/syb2.70052 (PMC12747248; doi:10.1049/syb2.70052)
Supplement: Supplementary file 2 — Table S2: The information of FAMRDEGs. [file SYB2-20-e70052-s002.docx]

Table S2. The information of FAMRDEGs

| gene | OR | OR_1 | OR_2 | P value | OR (95% CI) |
| --- | --- | --- | --- | --- | --- |
| PCTP | 2.136619 | 1.085104 | 3.419119 | 0.00028 | 2.14(1.09~3.42) |
| FABP3 | 4.404943 | 2.472835 | 6.916172 | 8.60E-05 | 4.4(2.47~6.92) |
| ABCD3 | 2.977044 | 1.450828 | 4.865957 | 0.00056 | 2.98(1.45~4.87) |
| ACSL4 | 1.962503 | 0.767501 | 3.405939 | 0.0035 | 1.96(0.77~3.41) |
| UCP3 | 1.812689 | 0.9247 | 2.956225 | 4.00E-04 | 1.81(0.92~2.96) |
| SLC27A6 | -1.2688 | -2.63636 | -0.01833 | 0.055 | -1.27(-2.64~-0.02) |
| CYP1B1 | -3.61709 | -5.64496 | -2.02688 | 7.50E-05 | -3.62(-5.64~-2.03) |
| ACBD4 | 2.17643 | 0.503856 | 4.086292 | 0.016 | 2.18(0.5~4.09) |
| CYP4B1 | -1.23894 | -2.09597 | -0.51401 | 0.0019 | -1.24(-2.1~-0.51) |
| TBXAS1 | -4.08753 | -6.52908 | -2.04648 | 0.00031 | -4.09(-6.53~-2.05) |
| CYP11B1 | 2.129636 | 0.155108 | 4.428847 | 0.048 | 2.13(0.16~4.43) |
| PPARA | 2.211487 | 0.859131 | 3.834005 | 0.0032 | 2.21(0.86~3.83) |
| CBR4 | -3.38639 | -5.34021 | -1.79315 | 0.00015 | -3.39(-5.34~-1.79) |
| ADRB2 | -4.2275 | -6.68195 | -2.26032 | 0.00015 | -4.23(-6.68~-2.26) |
| ALOX5AP | -1.91802 | -3.43514 | -0.69807 | 0.0054 | -1.92(-3.44~-0.7) |
| PHYH | 3.868741 | 1.563038 | 6.566575 | 0.0022 | 3.87(1.56~6.57) |
| PTGS1 | -2.07161 | -4.24892 | -0.10349 | 0.048 | -2.07(-4.25~-0.1) |
| IGF1 | 3.185069 | 1.702422 | 4.999234 | 0.00013 | 3.19(1.7~5) |
| SCP2 | 1.689083 | 0.473432 | 3.09198 | 0.011 | 1.69(0.47~3.09) |
| PECR | 5.776374 | 3.386987 | 8.914225 | 3.20E-05 | 5.78(3.39~8.91) |
| ACADM | 2.191599 | 0.946198 | 3.641455 | 0.0013 | 2.19(0.95~3.64) |
| EDN1 | -1.3564 | -2.38225 | -0.45929 | 0.0052 | -1.36(-2.38~-0.46) |
| RXRA | 1.520498 | 0.104872 | 3.091051 | 0.044 | 1.52(0.1~3.09) |
| PTGIS | -2.86652 | -4.84042 | -1.22379 | 0.0017 | -2.87(-4.84~-1.22) |
| LTC4S | -2.99964 | -5.06125 | -1.25295 | 0.0018 | -3(-5.06~-1.25) |
| PTGES3 | -1.45544 | -2.81821 | -0.30858 | 0.021 | -1.46(-2.82~-0.31) |
| FAS | 2.5226 | 0.427213 | 4.88954 | 0.025 | 2.52(0.43~4.89) |
| PLA2G4A | -1.46727 | -2.71121 | -0.34865 | 0.014 | -1.47(-2.71~-0.35) |
| SCAP | -1.82876 | -3.49524 | -0.33973 | 0.022 | -1.83(-3.5~-0.34) |
| DBI | 3.537755 | 1.638812 | 5.816653 | 0.00082 | 3.54(1.64~5.82) |
| PCCB | 2.775278 | 1.421082 | 4.482487 | 0.00034 | 2.78(1.42~4.48) |
| ALDH1B1 | 5.933132 | 3.530455 | 9.110574 | 2.30E-05 | 5.93(3.53~9.11) |
| LMNA | -2.75299 | -5.15659 | -0.70053 | 0.014 | -2.75(-5.16~-0.7) |
| SREBF2 | 5.940751 | 3.207777 | 9.401716 | 0.00014 | 5.94(3.21~9.4) |
| IDH1 | 7.492671 | 4.471509 | 11.55729 | 2.70E-05 | 7.49(4.47~11.56) |
| PPARGC1A | 3.144811 | 1.762803 | 5.038306 | 0.00013 | 3.14(1.76~5.04) |
| VEGFA | 3.156593 | 1.162295 | 5.536591 | 0.0043 | 3.16(1.16~5.54) |
| HPGD | -5.34716 | -8.22116 | -3.15508 | 2.70E-05 | -5.35(-8.22~-3.16) |
| PDK2 | 2.653009 | 0.951031 | 4.709225 | 0.0054 | 2.65(0.95~4.71) |
| SCD | -1.98333 | -3.2595 | -0.99788 | 0.00054 | -1.98(-3.26~-1) |
| HSD11B1 | -1.82146 | -2.98909 | -0.85756 | 0.00071 | -1.82(-2.99~-0.86) |
| MAPKAPK2 | 4.151822 | 1.885633 | 6.96617 | 0.0012 | 4.15(1.89~6.97) |
| PRKAG2 | 3.413028 | 1.200674 | 6.05329 | 0.0062 | 3.41(1.2~6.05) |
| SLC25A17 | -2.35178 | -4.38849 | -0.56388 | 0.015 | -2.35(-4.39~-0.56) |
| SCARB2 | -3.46478 | -5.66812 | -1.62433 | 0.00069 | -3.46(-5.67~-1.62) |
| APOO | 8.011665 | 4.955337 | 12.43614 | 1.70E-05 | 8.01(4.96~12.44) |
| SLC17A5 | -2.81303 | -4.79255 | -1.09463 | 0.0026 | -2.81(-4.79~-1.09) |
| FADS3 | -3.35502 | -6.06428 | -1.06201 | 0.0078 | -3.36(-6.06~-1.06) |
| ADH1B | -2.13821 | -3.35649 | -1.18098 | 9.90E-05 | -2.14(-3.36~-1.18) |
| ACOT8 | 3.145484 | 1.277575 | 5.426959 | 0.0027 | 3.15(1.28~5.43) |
| PDK1 | 2.991501 | 1.128744 | 5.14327 | 0.0032 | 2.99(1.13~5.14) |
| DECR2 | -2.28579 | -3.78306 | -1.03952 | 0.00098 | -2.29(-3.78~-1.04) |
| ADIPOR2 | 1.48733 | 0.033886 | 3.137799 | 0.057 | 1.49(0.03~3.14) |
| CS | 5.763616 | 3.213866 | 9.061704 | 8.90E-05 | 5.76(3.21~9.06) |
| ACLY | -2.44422 | -4.47275 | -0.67289 | 0.011 | -2.44(-4.47~-0.67) |
| ACACA | -3.59442 | -6.19436 | -1.38691 | 0.0031 | -3.59(-6.19~-1.39) |
| NDUFAB1 | 8.544211 | 4.810601 | 13.42854 | 8.60E-05 | 8.54(4.81~13.43) |
| PTGES2 | 5.179964 | 2.922353 | 8.051788 | 6.20E-05 | 5.18(2.92~8.05) |
| ACOT13 | 5.948892 | 3.506299 | 9.055211 | 2.20E-05 | 5.95(3.51~9.06) |
| FASN | -1.36195 | -2.75215 | -0.08403 | 0.043 | -1.36(-2.75~-0.08) |
| AMACR | 1.354844 | 0.205904 | 2.632678 | 0.027 | 1.35(0.21~2.63) |
| ACOX2 | 1.234076 | 0.449204 | 2.137255 | 0.0038 | 1.23(0.45~2.14) |
| ALOX5 | -1.64546 | -3.42843 | -0.11134 | 0.05 | -1.65(-3.43~-0.11) |
| CBR1 | 2.664075 | 1.314731 | 4.287521 | 0.00042 | 2.66(1.31~4.29) |
| CYP4F3 | -0.845 | -1.76294 | -0.01193 | 0.056 | -0.84(-1.76~-0.01) |
| DECR1 | 10.07683 | 5.89448 | 16.58399 | 0.00015 | 10.08(5.89~16.58) |
| DHCR7 | 2.917361 | 0.994023 | 5.198431 | 0.0061 | 2.92(0.99~5.2) |
| ETFA | 4.270863 | 2.105346 | 7.028941 | 6.00E-04 | 4.27(2.11~7.03) |
| ACSL1 | 2.26337 | 1.141846 | 3.677685 | 0.00039 | 2.26(1.14~3.68) |
| GCDH | 2.628031 | 0.447576 | 5.140293 | 0.026 | 2.63(0.45~5.14) |
| HADHB | 1.937862 | 0.611668 | 3.577575 | 0.01 | 1.94(0.61~3.58) |
| HSD17B4 | 2.805619 | 1.293683 | 4.6719 | 0.001 | 2.81(1.29~4.67) |
| PLIN1 | -0.89845 | -1.79405 | -0.18251 | 0.027 | -0.9(-1.79~-0.18) |
| SLC2A4 | 1.30737 | 0.37747 | 2.38933 | 0.0099 | 1.31(0.38~2.39) |
| AGT | -1.68532 | -3.42811 | -0.12986 | 0.043 | -1.69(-3.43~-0.13) |
| ALDH3A2 | 2.221962 | 0.478564 | 4.251585 | 0.02 | 2.22(0.48~4.25) |
| APOA1 | 1.527034 | 0.103591 | 3.128934 | 0.046 | 1.53(0.1~3.13) |
| APOE | -2.77533 | -4.6252 | -1.25377 | 0.0011 | -2.78(-4.63~-1.25) |
| C3 | -1.72893 | -2.76572 | -0.87287 | 0.00029 | -1.73(-2.77~-0.87) |
| CD36 | 0.945996 | 0.07704 | 1.923961 | 0.042 | 0.95(0.08~1.92) |
| FAAH | -2.64805 | -4.2604 | -1.30881 | 0.00037 | -2.65(-4.26~-1.31) |
| FABP1 | 2.029065 | 0.247709 | 4.017944 | 0.033 | 2.03(0.25~4.02) |
| FABP5 | 1.018741 | 0.133616 | 2.020575 | 0.032 | 1.02(0.13~2.02) |
| LEP | -2.89979 | -5.15814 | -1.15579 | 0.0045 | -2.9(-5.16~-1.16) |
| PCCA | 3.186424 | 1.654054 | 5.087954 | 0.00024 | 3.19(1.65~5.09) |
| ICAM1 | 1.593004 | 0.048684 | 3.306683 | 0.052 | 1.59(0.05~3.31) |
| ECI2 | 5.231075 | 3.073678 | 8.047253 | 2.90E-05 | 5.23(3.07~8.05) |
| LPXN | -2.28817 | -4.37266 | -0.4654 | 0.02 | -2.29(-4.37~-0.47) |
| AKT1 | 1.701589 | 0.327546 | 3.601551 | 0.043 | 1.7(0.33~3.6) |
| HCCS | 4.753101 | 2.698094 | 7.413417 | 6.10E-05 | 4.75(2.7~7.41) |
| ACTB | -2.97829 | -4.7818 | -1.60426 | 0.00018 | -2.98(-4.78~-1.6) |
| IRS1 | 4.671915 | 2.795241 | 7.255405 | 3.00E-05 | 4.67(2.8~7.26) |
| ACAA2 | 2.83449 | 1.204215 | 4.837485 | 0.002 | 2.83(1.2~4.84) |
| LGALS1 | -3.7483 | -5.77503 | -2.11857 | 4.70E-05 | -3.75(-5.78~-2.12) |
| SLC25A20 | 3.762302 | 1.743356 | 6.241152 | 0.00097 | 3.76(1.74~6.24) |
| ACAA1 | 2.835554 | 0.764214 | 5.400293 | 0.016 | 2.84(0.76~5.4) |
| COG2 | 3.854826 | 1.807494 | 6.333724 | 0.00073 | 3.85(1.81~6.33) |
| MLYCD | 0.888622 | 0.009855 | 1.898225 | 0.061 | 0.89(0.01~1.9) |
| ABCD1 | 2.759143 | 0.63734 | 5.160756 | 0.016 | 2.76(0.64~5.16) |
| OPN3 | 6.731165 | 4.106142 | 10.12537 | 8.90E-06 | 6.73(4.11~10.13) |
| HPGDS | -1.40489 | -2.58957 | -0.36762 | 0.012 | -1.4(-2.59~-0.37) |
| MORC2 | -2.31448 | -4.2201 | -0.68671 | 0.0094 | -2.31(-4.22~-0.69) |
| HSD17B7 | -4.92027 | -7.51743 | -2.91853 | 2.10E-05 | -4.92(-7.52~-2.92) |
| MECR | 1.798955 | 0.392719 | 3.422032 | 0.018 | 1.8(0.39~3.42) |
| HSD17B12 | 1.785399 | 0.371234 | 3.384091 | 0.019 | 1.79(0.37~3.38) |
| RBP4 | -1.02582 | -2.1903 | -0.15809 | 0.047 | -1.03(-2.19~-0.16) |
| OLAH | -2.06654 | -3.35821 | -0.98819 | 0.00055 | -2.07(-3.36~-0.99) |
| CA4 | 1.910656 | 0.871034 | 3.226293 | 0.0013 | 1.91(0.87~3.23) |
| ABCC1 | 3.537079 | 1.560336 | 5.857088 | 0.0011 | 3.54(1.56~5.86) |

FAMRDEGs: fatty acid metabolism related differentially expressed genes
