# Supplementary material for: Exploring and Validating the Molecular Mechanisms Linking Fatty Acid Metabolism and Sarcopenia
Source: IET Syst Biol. 2025 Dec 29;20(1):e70052. doi: 10.1049/syb2.70052 (PMC12747248; doi:10.1049/syb2.70052)
Supplement: Supplementary file 3 — Table S3: The network relations of 7 key genes and 54 miRNAs. [file SYB2-20-e70052-s003.docx]

Table S3. The network relations of 7 key genes and 54 miRNAs

| mRNA | miRNA |
| --- | --- |
| OPN3 | hsa-miR-101-3p |
| OPN3 | hsa-miR-130a-3p |
| OPN3 | hsa-miR-32-3p |
| OPN3 | hsa-miR-3662 |
| OPN3 | hsa-miR-501-3p |
| ACOT8 | hsa-miR-130a-3p |
| ACOT8 | hsa-miR-130b-3p |
| ACOT8 | hsa-miR-301a-3p |
| ACOT8 | hsa-miR-301b-3p |
| ACOT8 | hsa-miR-424-5p |
| DECR1 | hsa-miR-128-3p |
| DECR1 | hsa-miR-148a-3p |
| DECR1 | hsa-miR-148b-3p |
| DECR1 | hsa-miR-152-3p |
| DECR1 | hsa-miR-190a-5p |
| DECR1 | hsa-miR-190b |
| DECR1 | hsa-miR-19b-1-5p |
| DECR1 | hsa-miR-32-3p |
| DECR1 | hsa-miR-34b-3p |
| PPARGC1A | hsa-miR-27a-3p |
| PPARGC1A | hsa-miR-27b-3p |
| PPARGC1A | hsa-miR-520a-3p |
| PPARGC1A | hsa-miR-520c-3p |
| PECR | hsa-miR-124-3p |
| PCTP | hsa-miR-106b-5p |
| PCTP | hsa-miR-125a-5p |
| PCTP | hsa-miR-125b-5p |
| PCTP | hsa-miR-132-3p |
| PCTP | hsa-miR-20a-5p |
| PCTP | hsa-miR-605-5p |
| SREBF2 | hsa-miR-10a-3p |
| SREBF2 | hsa-miR-149-5p |
| SREBF2 | hsa-miR-151a-5p |
| SREBF2 | hsa-miR-16-5p |
| SREBF2 | hsa-miR-181a-5p |
| SREBF2 | hsa-miR-1915-3p |
| SREBF2 | hsa-miR-193b-3p |
| SREBF2 | hsa-miR-20a-3p |
| SREBF2 | hsa-miR-21-5p |
| SREBF2 | hsa-miR-221-3p |
| SREBF2 | hsa-miR-221-5p |
| SREBF2 | hsa-miR-24-3p |
| SREBF2 | hsa-miR-29a-3p |
| SREBF2 | hsa-miR-29b-1-5p |
| SREBF2 | hsa-miR-30c-1-3p |
| SREBF2 | hsa-miR-335-5p |
| SREBF2 | hsa-miR-424-5p |
| SREBF2 | hsa-miR-4690-5p |
| SREBF2 | hsa-miR-489-3p |
| SREBF2 | hsa-miR-509-3p |
| SREBF2 | hsa-miR-542-3p |
| SREBF2 | hsa-miR-650 |
| SREBF2 | hsa-miR-766-3p |
| SREBF2 | hsa-let-7e-5p |
| SREBF2 | hsa-miR-185-5p |
| SREBF2 | hsa-miR-4701-3p |
| SREBF2 | hsa-miR-342-3p |
