# Supplementary material for: Exploring and Validating the Molecular Mechanisms Linking Fatty Acid Metabolism and Sarcopenia
Source: IET Syst Biol. 2025 Dec 29;20(1):e70052. doi: 10.1049/syb2.70052 (PMC12747248; doi:10.1049/syb2.70052)
Supplement: Supplementary file 4 — Table S4: The network relations of 9 key genes and 102 TFs. [file SYB2-20-e70052-s001.docx]

Table S4. The network relations of 9 key genes and 102 TFs

| mRNA | TF |
| --- | --- |
| ACOT8 | ARID2 |
| ACOT8 | BCL11A |
| ACOT8 | BRD2 |
| ACOT8 | CEBPB |
| ACOT8 | EBF1 |
| ACOT8 | EGR1 |
| ACOT8 | ELF1 |
| ACOT8 | ELK1 |
| ACOT8 | EP300 |
| ACOT8 | ERG |
| ACOT8 | ESR1 |
| ACOT8 | ESRRA |
| ACOT8 | ETS1 |
| ACOT8 | ETV1 |
| ACOT8 | FLI1 |
| ACOT8 | FOS |
| ACOT8 | FOSL1 |
| ACOT8 | FOSL2 |
| ACOT8 | FOXA1 |
| ACOT8 | FOXA2 |
| ACOT8 | GABPA |
| ACOT8 | GATA1 |
| ACOT8 | GATA2 |
| ACOT8 | GATA3 |
| ACOT8 | GRHL2 |
| ACOT8 | HDAC1 |
| ACOT8 | HNF4A |
| ACOT8 | IKZF1 |
| ACOT8 | IRF3 |
| ACOT8 | IRF4 |
| ACOT8 | JUN |
| ACOT8 | JUNB |
| ACOT8 | JUND |
| ACOT8 | MAFK |
| ACOT8 | MAX |
| ACOT8 | MAZ |
| ACOT8 | MED1 |
| ACOT8 | NELFA |
| ACOT8 | NFYA |
| ACOT8 | NFYB |
| ACOT8 | NOTCH1 |
| ACOT8 | NR2F1 |
| ACOT8 | NR2F2 |
| ACOT8 | NRF1 |
| ACOT8 | POLR2A |
| ACOT8 | REST |
| ACOT8 | RFX5 |
| ACOT8 | RUNX1 |
| ACOT8 | RUNX1T1 |
| ACOT8 | RXRA |
| ACOT8 | SMAD3 |
| ACOT8 | SMAD4 |
| ACOT8 | SMARCA4 |
| ACOT8 | SMARCC1 |
| ACOT8 | SP1 |
| ACOT8 | SP2 |
| ACOT8 | SRF |
| ACOT8 | STAT3 |
| ACOT8 | SUPT5H |
| ACOT8 | TAL1 |
| ACOT8 | TBP |
| ACOT8 | TEAD4 |
| ACOT8 | TFAP2A |
| ACOT8 | TFAP2C |
| ACOT8 | YY1 |
| DECR1 | ELF1 |
| DECR1 | ELK4 |
| DECR1 | ERG |
| DECR1 | ESRRA |
| DECR1 | ETS1 |
| DECR1 | ETV1 |
| DECR1 | FOXA1 |
| DECR1 | FOXA2 |
| DECR1 | GABPA |
| DECR1 | HNF4A |
| DECR1 | NRF1 |
| DECR1 | POLR2A |
| DECR1 | RXRA |
| DECR1 | TBP |
| FABP3 | ARNT |
| FABP3 | BHLHE40 |
| FABP3 | CEBPA |
| FABP3 | CEBPB |
| FABP3 | CTCF |
| FABP3 | E2F6 |
| FABP3 | ESR1 |
| FABP3 | ESRRA |
| FABP3 | FLI1 |
| FABP3 | GATA2 |
| FABP3 | GRHL2 |
| FABP3 | MAX |
| FABP3 | MYC |
| FABP3 | MYOD1 |
| FABP3 | NR2F2 |
| FABP3 | RAD21 |
| FABP3 | REST |
| FABP3 | RXRA |
| FABP3 | SMARCA4 |
| FABP3 | SNAI2 |
| FABP3 | SPI1 |
| FABP3 | STAT3 |
| FABP3 | TEAD1 |
| FABP3 | TEAD4 |
| FABP3 | TFAP2A |
| FABP3 | ZNF24 |
| HSD17B7 | CEBPB |
| HSD17B7 | CREB1 |
| HSD17B7 | CTCF |
| HSD17B7 | ELF1 |
| HSD17B7 | MAX |
| HSD17B7 | USF1 |
| HSD17B7 | USF2 |
| OPN3 | ESR1 |
| OPN3 | FOS |
| OPN3 | FOSL2 |
| OPN3 | FOXA1 |
| OPN3 | FOXA2 |
| OPN3 | HES2 |
| OPN3 | HNF4A |
| OPN3 | JUN |
| OPN3 | JUND |
| OPN3 | MAFK |
| OPN3 | MAX |
| OPN3 | MYC |
| OPN3 | NFE2L2 |
| OPN3 | RUNX1 |
| OPN3 | SP1 |
| OPN3 | STAT3 |
| OPN3 | TBP |
| OPN3 | TEAD1 |
| OPN3 | TEAD4 |
| OPN3 | CEBPA |
| OPN3 | CEBPB |
| OPN3 | ARID1A |
| OPN3 | CREB1 |
| OPN3 | CTCF |
| PCTP | CTCF |
| PCTP | CTCFL |
| PCTP | ELF1 |
| PCTP | ERG |
| PCTP | ESR1 |
| PCTP | ETS1 |
| PCTP | ETV1 |
| PCTP | GABPA |
| PCTP | NELFA |
| PCTP | NRF1 |
| PCTP | RAD21 |
| PCTP | SMC3 |
| PCTP | STAT3 |
| PCTP | TFAP2A |
| PCTP | BRD4 |
| PECR | ATF2 |
| PECR | BRD3 |
| PECR | CEBPA |
| PECR | CEBPB |
| PECR | CTCF |
| PECR | E2F1 |
| PECR | E2F4 |
| PECR | ELF1 |
| PECR | ELK1 |
| PECR | ELK4 |
| PECR | EP300 |
| PECR | ERG |
| PECR | ETS1 |
| PECR | ETV1 |
| PECR | FLI1 |
| PECR | FOXA1 |
| PECR | GABPA |
| PECR | GATA2 |
| PECR | HNF4A |
| PECR | KMT2A |
| PECR | MAX |
| PECR | MITF |
| PECR | NELFA |
| PECR | NRF1 |
| PECR | POLR2A |
| PECR | RAD21 |
| PECR | RELA |
| PECR | REST |
| PECR | SMARCA4 |
| PECR | SPI1 |
| PECR | STAG1 |
| PECR | STAT3 |
| PECR | SUPT5H |
| PECR | TBP |
| PECR | TEAD4 |
| PECR | USF1 |
| PECR | USF2 |
| PECR | ZEB1 |
| PECR | ZNF384 |
| PPARGC1A | AR |
| PPARGC1A | ATF2 |
| PPARGC1A | FOXA1 |
| PPARGC1A | FOXA2 |
| PPARGC1A | MAX |
| PPARGC1A | USF1 |
| PPARGC1A | USF2 |
| SREBF2 | ATF2 |
| SREBF2 | CEBPB |
| SREBF2 | CHD4 |
| SREBF2 | E2F1 |
| SREBF2 | EGR1 |
| SREBF2 | ESRRA |
| SREBF2 | KMT2A |
| SREBF2 | NFKB1 |
| SREBF2 | NFYA |
| SREBF2 | NRF1 |
| SREBF2 | SMAD3 |
| SREBF2 | SP1 |
| SREBF2 | TCF12 |
| SREBF2 | TFAP4 |
| SREBF2 | YY1 |

TFs: transcription factors
